# Willingness to Know the Cause of Death and Hypothetical Acceptability of the Minimally Invasive Autopsy in Six Diverse African and Asian Settings: A Mixed Methods Socio-Behavioural Study

**DOI:** 10.1371/journal.pmed.1002172

**Published:** 2016-11-22

**Authors:** Maria Maixenchs, Rui Anselmo, Emily Zielinski-Gutiérrez, Frank O. Odhiambo, Clarah Akello, Maureen Ondire, S. Shujaat H. Zaidi, Sajid Bashir Soofi, Zulfiqar A. Bhutta, Kounandji Diarra, Mahamane Djitèye, Roukiatou Dembélé, Samba Sow, Pamela Cathérine Angoissa Minsoko, Selidji Todagbe Agnandji, Bertrand Lell, Mamudo R. Ismail, Carla Carrilho, Jaume Ordi, Clara Menéndez, Quique Bassat, Khátia Munguambe

**Affiliations:** 1 Centro de Investigação em Saúde da Manhiça, Maputo, Mozambique; 2 ISGlobal, Barcelona Centre for International Health Research (CRESIB), Hospital Clínic de Barcelona, Universitat de Barcelona, Barcelona, Spain; 3 Centers for Disease Control and Prevention (CDC-Kenya), Nairobi, Kenya; 4 Kenya Medical Research Institute, Centre for Global Health Research, Kisumu, Kenya; 5 Centre of Excellence in Women and Child Health, Aga Khan University, Karachi, Pakistan; 6 Centre for Global Child Health, Hospital for Sick Children, Toronto, Ontario, Canada; 7 Centre pour le Développement des Vaccins (CVD-Mali), Bamako, Mali; 8 Centre de Recherches Médicales de Lambaréné, Hôpital Albert Schweitzer, Lambaréné, Gabon; 9 Institut für Tropenmedizin, Universitätsklinikum Tübingen, Tübingen, Germany; 10 Department of Pathology, Maputo Central Hospital, Maputo, Mozambique; 11 Catalan Institution for Research and Advanced Studies (ICREA), Barcelona, Spain; 12 Faculty of Medicine, Eduardo Mondlane University, Maputo, Mozambique; Umeå Centre for Global Health Research, Umeå University, SWEDEN

## Abstract

**Background:**

The minimally invasive autopsy (MIA) is being investigated as an alternative to complete diagnostic autopsies for cause of death (CoD) investigation. Before potential implementation of the MIA in settings where post-mortem procedures are unusual, a thorough assessment of its feasibility and acceptability is essential.

**Methods and Findings:**

We conducted a socio-behavioural study at the community level to understand local attitudes and perceptions related to death and the hypothetical feasibility and acceptability of conducting MIAs in six distinct settings in Gabon, Kenya, Mali, Mozambique, and Pakistan. A total of 504 interviews (135 key informants, 175 health providers [including formal health professionals and traditional or informal health providers], and 194 relatives of deceased people) were conducted. The constructs “willingness to know the CoD” and “hypothetical acceptability of MIAs” were quantified and analysed using the framework analysis approach to compare the occurrence of themes related to acceptability across participants.

Overall, 75% (379/504) of the participants would be willing to know the CoD of a relative. The overall hypothetical acceptability of MIA on a relative was 73% (366/504). The idea of the MIA was acceptable because of its perceived simplicity and rapidity and particularly for not “mutilating” the body. Further, MIAs were believed to help prevent infectious diseases, address hereditary diseases, clarify the CoD, and avoid witchcraft accusations and conflicts within families. The main concerns regarding the procedure included the potential breach of confidentiality on the CoD, the misperception of organ removal, and the incompatibility with some religious beliefs. Formal health professionals were concerned about possible contradictions between the MIA findings and the clinical pre-mortem diagnoses. Acceptability of the MIA was equally high among Christian and Islamic communities. However, in the two predominantly Muslim countries, MIA acceptability was higher in Mali than in Pakistan.

While the results of the study are encouraging for the potential use of the MIA for CoD investigation in low-income settings, they remain hypothetical, with a need for confirmation with real-life MIA implementation and in populations beyond Health and Demographic Surveillance System areas.

**Conclusions:**

This study showed a high level of interest in knowing the CoD of a relative and a high hypothetical acceptability of MIAs as a tool for CoD investigation across six distinct settings. These findings anticipate potential barriers and facilitators, both at the health facility and community level, essential for local tailoring of recommendations for future MIA implementation.

## Abstract 2

Using a mixed methods socio-behavioral approach, Maria Maixenchs and colleagues investigate the willingness to know the cause of death and acceptability of the Minimally Invasive Autopsy in Gabon, Kenya, Mali, Mozambique and Pakistan.

## Author Summary

**Why Was This Study Done?:**

- Reliable data on the real causes of death in low- and middle-income countries are scarce. Without this information, it is difficult to focus on the real health problems of a population and to implement adequate health policies.
- Minimally invasive autopsies are being studied as a new method to determine the cause of death.

**What Did the Researchers Do and Find?:**

- A total of 504 interviews were conducted in five different countries to investigate the willingness of individuals to know the cause of death of a relative and the hypothetical acceptability of minimally invasive autopsies.
- Willingness to know the cause of death was high, as was the hypothetical acceptability of this innovative method to investigate cause of death.

**What Do These Findings Mean?:**

- These results are encouraging because they open new research lines for investigating the cause of death in areas where data are difficult to obtain and general information on mortality is necessary to design effective preventive policies.

## Introduction

Current estimates of causes of mortality in low- and middle-income countries are hampered by the lack of reliable data. In these regions, the feasibility of routinely conducting the complete diagnostic autopsy (CDA), considered the gold standard methodology for cause of death (CoD) investigation, faces notable—and often insurmountable—barriers. These include, among others, the poor or non-existent acceptability of the typical and highly disfiguring CDA procedure, the lack of pathology expertise and infrastructure in low-resource regions, and the fact that in such settings the majority of deaths occur outside of formal health system premises [1,2]. The practice of the CDA is therefore restricted to research projects, forensic investigations for medico-legal purposes, and deaths reaching referral hospitals in the few countries that have functional pathology services capable of routinely performing autopsies [3–5]. As a consequence of such challenges, the use of less invasive post-mortem sampling techniques, such as the minimally invasive autopsy (MIA), is being considered as a potential substitute for the CDA [6–8]. The MIA consists of a series of post-mortem punctures using fine biopsy needles aiming to obtain tissue samples and body fluids from a corpse within the first hours after death, which are then submitted for a thorough histopathological and microbiological investigation of the underlying CoD. A multicentre study has recently been concluded to validate the MIA tool in direct comparison with the CDA for CoD investigation. Preliminary results show good evidence that the quantity and quality of the samples obtained is sufficient and valuable for CoD investigation and a good diagnostic concordance between both methods for all age groups [9]. The use of this approach is expected to be more acceptable, compared to the CDA, because it is non-disfiguring, performed quickly, and technically much more feasible (simple and easy to perform by minimally trained personnel). Although scarce, the literature on the MIA’s acceptability suggests that willingness to know the CoD, interest in prevention of future deaths from similar causes, and learning more about the disease to which the death has been attributed are some of the factors that may promote its acceptance [10,11].

Nevertheless, before any attempt to implement MIAs in areas where post-mortem procedures are unusual, it is critical to understand attitudes and perceptions in relation to death and the manipulation of bodies, as well as the potential barriers and challenges to the implementation of such methods. In an effort to further understand these issues, and in prevision of the potential utilisation of such a tool for CoD investigation in some of the participating sites, we conducted a socio-behavioural study aiming to explore the willingness to know the CoD (regardless of the method used to establish the CoD) and the feasibility and hypothetical acceptability of the MIA in six distinct geographical, cultural, and religious backgrounds in Africa and Asia, namely, Gabon, Kenya, Mali, Mozambique, and Pakistan.

## Methods

The study was approved centrally by the Clinical Research Ethics Committee of the Hospital Clínic de Barcelona, Spain, and locally by the following institutional review boards and ethics committees at each site: the ethics committee of the Faculté de Médecine, Pharmacologie et Odonto-Stomatologie of the Université de Bamako (Mali); the Comité d’Éthique Régional Indépendant de Lambaréné (Gabon); Kenya Medical Research Institute local and national scientific steering committees and national ethical review committees (Kenya); the Manhiça Health Research Centre (Centro de Investigação em Saúde da Manhiça) institutional bioethics committee (CIBS-CISM) and the National Committee for Bioethics in Health (Comité Nacional de Bioética para Saúde) (Mozambique); and the Aga Khan University Ethics Review Committee and the National Research Ethics Committee of the Government of Pakistan (Pakistan). Written informed consent was obtained from all participants. In the case of illiteracy, participants could provide a thumbprint that would be countersigned by an impartial witness, guaranteeing that participation had been voluntary. All data were managed based on unique identification numbers so as to guarantee the respondent’s confidentiality.

This study was part of a project designed to validate a new MIA protocol for CoD investigation in low- and middle-income countries that incorporated a comprehensive ethnographic study with the main objective of understanding local attitudes and perceptions related to death at the community level and the feasibility and acceptability of conducting MIAs in deaths occurring both within and outside formal health service premises. The ethnographic study was conducted between September 2013 and July 2015 in six sites in the following countries: Gabon, Kenya, Mali, Mozambique, and Pakistan. A diversity of contexts were selected, which included urban, semi-urban, and rural areas. A common protocol and methodology were used in all countries. Although the study design was qualitative, the present article focuses on a descriptive analysis performed for all quantifiable constructs that emerged from the main themes that surfaced from the qualitative data. This study did not have a predefined written prospective analytical plan, but the analyses being reported were decided prior to data collection, although some of the preliminary results contributed to the further examination of emerging themes.

### Study Settings

The six study settings were chosen so as to provide a wide representation of different cultural, religious, geographical, and epidemiological backgrounds within low- and middle-income countries in Africa and Asia.

#### Lambaréné (Gabon)

In Gabon, the study was conducted in Lambaréné, the seventh largest city in Gabon, within a predominantly semi-urban area. Its population of approximately 70,000 inhabitants participates in a Health and Demographic Surveillance System (HDSS) established by the Medical Research Centre of Lambaréné (Centre de Recherches Médicales de Lambaréné). Two district hospitals, two healthcare centres, and ten dispensaries provide health services in the area. CDAs are not routinely conducted in any of these health facilities. People residing in Lambaréné are from a diversity of cultural and ethnic backgrounds, reflecting what is found in the rest of the country, where the most dominant and ancient ethnic groups are the Omyene, Fangs, Ghischira, and Tsogo. Gabon is a non-clerical country where three main religious and traditional belief systems coexist, namely, Animism, Christianity, and Islam.

#### Kisumu (Kenya)

In Kenya, the study took place in Siaya County, in western Kenya, nearby Kisumu, the third largest city in the country. The area is under a HDSS run by the Kenya Medical Research Council and CDC-Kenya that covers approximately 227,400 inhabitants. The health service network in the area consists of two regional hospitals and over 30 health centres and dispensaries. CDAs can be conducted at the regional hospitals, though they are rarely performed. The majority of Siaya County residents are of the Luo ethnic group. Christianity is the predominant religion, though Muslims are also found, and adherence to traditional belief systems is also common.

#### Bamako (Mali)

In Mali, the study took place in the Banconi and Djicoroni districts in Bamako, Mali’s capital. In this area, a HDSS was established by the Centre pour le Développement des Vaccins (CVD-Mali), covering a population of around 2 million inhabitants. A tertiary-level teaching hospital and three health centres provide the health services in the area. Pathology facilities are available, but autopsies are very seldom conducted. Among the ten main ethnic groups in the study area, the Bambara, the Fulani, the Sonrais, and the Soninke are the largest ones. The majority of the people are Muslims, and there is a minority of Christians and Animists.

#### Maputo and Manhiça (Mozambique)

In Mozambique, the study took place in Maputo, the country’s capital, and in Manhiça District, 80 km north of Maputo. Maputo is an urban centre with a population of approximately 2 million people, with the majority living in semi-urban areas. The city is served by one central hospital, where CDAs are routinely conducted, as well as three general hospitals and several health centres. There is a mix of ethnic, religious, and social backgrounds among its population, with Christians and Animists being the most predominant groups, while Muslims represent a minority of the population. Manhiça District is covered by the Manhiça Health Research Centre HDSS, with approximately 160,000 inhabitants living in a predominantly rural area. Health services in the district are provided by a district hospital, a rural hospital, and 12 health centres, none with the capacity to conduct post-mortem procedures. Shangaans constitute the dominant ethnic group, with very strong patriarchal social structures and cultural aspects that are similar to those of other ethnic groups within the southern region of Africa. Animism and Christianity are the main religious/belief systems in this area, with a very small minority of Muslims.

#### Karachi (Pakistan)

The study in Pakistan was carried out in the port city of Karachi, with an estimated population of 25.9 million people. The study involved 13 towns of Karachi, with a population of approximately 20.2 million, served by seven teaching hospitals, three tertiary-level hospitals, and numerous clinics and health centres. Although pathology departments are in place in several of these hospitals, routine post-mortem procedures are seldom performed outside of forensic indications. The vast majority of the population is Muslim, but traditional beliefs are also common practice.

### Study Participants

The target population for this study comprised people who might best be able to describe the phenomenon of death because they (i) have been part of it through losing a relative, (ii) have been affected by it, or (iii) could affect or influence those who are part of it. The study targeted three groups, namely, key informants, health providers (formal health professionals and informal health providers), and relatives of deceased persons. Table 1 describes the number of people involved in each of the target groups.

**Table 1:** Number of interviews by target group and country.

Participants	Country					Total
	Gabon	Kenya	Mali	Mozambique	Pakistan	
**Key informants**	26	30	23	27	29	**135**
**Health providers**	29	45	37	33	31	**175**
**Relatives of deceased people**	29	54	31	46	34	**194**
***Relatives of deceased people who experienced a death within 24 h of the interview*** *	4	4	2	9	5	**24**
Maternal death (pregnant women)	0	4	0	0	1	**5**
Stillbirth/newborn death	1	0	0	2	0	**3**
Child death (>1 mo–14 y old)	0	0	0	2	0	**2**
Adult death (15–60 y old)	2	0	1	4	2	**9**
Elder death (>60 y old)	1	0	1	1	2	**5**
***Relatives of deceased people who experienced a death in the preceding 1–7 d*** *	9	17	14	25	12	**77**
Maternal death (pregnant women)	2	1	2	2	3	**10**
Stillbirth/newborn death	0	3	0	4	1	**8**
Child death (>1 mo–14 y old)	0	2	4	4	2	**12**
Adult death (15–60 y old)	2	6	3	11	3	**25**
Elder death (>60 y old)	5	5	5	4	3	**22**
***Relatives of deceased people who experienced a death in the preceding 30–40 d*** *	16	33	15	12	17	**93**
Maternal death (pregnant women)	0	6	1	1	3	**11**
Stillbirth/newborn death	1	5	1	3	1	**11**
Child death (>1 mo–14 y old)	0	2	2	2	1	**7**
Adult death (15–60 y old)	9	10	6	3	6	**34**
Elder death (>60 y old)	6	10	5	3	6	**30**
**Total**						**504**

*Groups are independent and therefore not cumulative.

Key informants comprised (i) people who are very familiar with the way of life of the communities involved in the study and/or can influence them regarding how to face, deal with, or react to the phenomenon of death (e.g., local political and traditional authorities, religious leaders, teachers), (ii) people who are very familiar with local cultural and religious norms and requirements for death-related events (e.g., community and religious leaders, funeral home personnel, body washers), and (iii) people who formulate and/or implement guidelines and codes of conduct related to health, disease, and death at the local level (e.g., policy makers, governmental authorities).

In this study, health providers comprised those who, through the nature of their work, are likely to be in contact with bodies at the time of death and likely to interact with grieving families within the first moments of the occurrence of a death. This group included nurses, midwives, lady health visitors, community health workers, clinicians, physicians, pathologists, traditional birth attendants, and traditional healers.

Relatives were defined as the closest possible persons to the deceased, although not necessary legally related. Three groups were interviewed according to the time of death: (i) those who experienced a death in the preceding 24 h, (ii) those who experienced a death in the previous 1–7 d, and (iii) those who experienced a death in the preceding 30–40 d.

Those who were not willing to talk about their experiences, were younger than 18 y of age, were not able to provide signed informed consent, and, among the relatives group, whose relative’s death was accidental or a violent one, were excluded from the study.

### Study Procedures

Data were collected by social scientists and research assistants, through in-depth interviews with key informants, semi-structured interviews with health providers, and interviews and informal conversations with relatives of deceased persons.

Training of the socio-anthropological team was conducted in a centralised manner during the investigators meeting held in Maputo in 2013, prior to study initiation. The MIA definition, which was standardised across sites, was “targeted small diagnostic biopsies (by needle puncture) of key organs, with or without the need of any supporting imaging technique”. The definition of MIA was then translated into the local language, and appropriate context-specific terminology was adapted to capture this concept. Visual aids of the needles and the types of samples obtained with them were produced to help field workers explain the procedure in the field.

Prior to data collection, meetings with community and religious leaders and local associations (women, youth, and community-based cooperatives) were held to explain the study and to seek advice about appropriate ways to approach community members in general and mourning relatives in particular. In Karachi these meetings also involved high-level entities such as government officers, non-governmental organisation representatives, and academia.

In Manhiça and Bamako, key informants were recruited during the above-mentioned meetings, while in Kisumu and Lambaréné they were identified individually in their residences or workplaces. In Karachi, focal persons identified at the institutional level (universities, colleges, and religious madrasahs, among others) assisted the team in identifying eligible key respondents.

Study research assistants recruited formal health professionals at health facilities. Traditional and informal community healthcare providers were identified and recruited through the same system established for identifying key informants.

Different channels were used to identify relatives of recently deceased individuals in the health facilities and in the communities. In Maputo and Bamako, social scientists or research assistants directly contacted the relatives of deceased persons at morgues, at hospitals, and in the community; in Manhiça, Lambaréné, Kisumu, and Karachi, funeral home caretakers, health facility staff, HDSS field workers, community leaders, village reporters, or community focal persons (liaisons with the research teams) continuously notified recent deaths to research assistants, who in turn contacted the relatives as soon as possible after notification for interviews.

All data were digitally recorded and transcribed verbatim, locally at each site. When permission for recording was not granted, detailed notes were taken. When interviews were conducted in the local language, transcripts were translated into the official language of the respective country (English, Portuguese, or French).

Data management and data analysis were conducted locally at each site. Centralised training was conducted during the investigators meeting prior to the initiation of activities. Activities were supervised by the social sciences principal investigator during the data collection and analysis processes. Three additional face-to-face meetings of investigators (Manhiça, Mozambique, 2013; Lambaréné, Gabon, 2014; Barcelona, Spain, 2015) were also utilised to retrain and refine the analytical strategy and skills, prioritising sites with less experience in qualitative data collection and analysis. Qualitative data underwent content analysis using the framework approach [12,13], whereby transcriptions were summarised and tabulated into a matrix format using MS Excel. This tabulation allowed data to be synthesised into two major concepts determined in advance of data entry: (i) willingness to know the CoD and (ii) hypothetical acceptability of conducting the MIA. Data regarding these two concepts were further reduced into three possible categories: “yes”, “no”, and “only under certain circumstances”, which were summarised into frequency distribution tables. The two concepts were cross-tabulated with study area and the socio-demographic characteristics of the respondents and the deceased person.

Further concepts that are not straightforwardly quantifiable—namely, perceived advantages of CoD determination, perceived disadvantages of CoD determination, facilitators for the MIA, and barriers to MIA—underwent data reduction whereby the different answers given by the respondents were grouped into general themes (thematic analysis).

## Results

A total of 504 interviews (135 key informants, 175 health providers, and 194 relatives of deceased persons) were conducted. Sixty-three percent of the people interviewed were male. Most participants were between 30 and 50 y of age. Most respondents were Christian (280/504, 56%) or Muslim (189/504, 37%). Detailed socio-demographic characteristics of the study participants are shown in Table 2. Twenty-eight people were approached but refused to participate, all of them among the relatives of deceased people group. Reasons for non-participation were mainly related to the state of mind around the death being incompatible with being interviewed (15 persons), with four of them indicating that they were too resentful to talk about their experience. Six persons refused to talk because they had no time, two because they had to travel, two asked for money in exchange for their time, and three people did not give any reason.

**Table 2:** Socio-demographic characteristics of participants.

Characteristic	Gabon: Lambaréné (Semi-urban), *n* = 84	Kenya: Kisumu (Rural), *n* = 129	Mali: Bamako (Urban), *n* = 91	Mozambique		Pakistan: Karachi (Urban), *n* = 94	Total, *n* = 504
				Manhiça (Rural), *n* = 81	Maputo (Urban), *n* = 25		
**Participants by site (percent)**	16.7	25.6	18.1	16.1	5.0	18.7	100.0
**Gender**							
Male	63 (75.0)	71 (55.0)	64 (70.3)	38 (46.9)	17 (68.0)	63 (67.0)	316 (62.7)
Female	21 (25.0)	58 (45.0)	27 (29.7)	43 (53.1)	8 (32.0)	31 (33.0)	188 (37.3)
**Age**							
18–29 y	5 (6.0)	29 (22.5)	7 (87.7)	8 (9.9)	9 (36.0)	25 (26.6)	83 (16.5)
30–49 y	43 (51.2)	58 (45.0)	39 (42.9)	29 (35.8)	12 (48.0)	55 (58.5)	236 (46.8)
>50 y	36 (42.9)	42 (32.6)	45 (49.5)	44 (54.3)	4 (16.0)	14 (14.9)	185 (36.5)
**Education**							
No schooling	6 (7.1)	14 (10.9)	11 (12.1)	18 (22.2)	1 (4.0)	9 (9.6)	59 (11.7)
Primary	13 (15.5)	48 (37.2)	16 (17.6)	41 (50.6)	8 (32.0)	7 (7.4)	133 (26.4)
Secondary	31 (36.9)	20 (15.5)	13 (14.3)	7 (8.6)	1 (4.0)	19 (20.2)	91 (18.1)
Quranic school	0 (0.0)	0 (0.0)	22 (24.2)	0 (0.0)	0 (0.0)	0 (0.0)	22 (4.4)
Professional training	13 (15.5)	28 (21.7)	2 (2.2)	12 (14.8)	3 (12.0)	20 (21.3)	78 (15.5)
University or higher	21 (25.0)	19 (14.7)	27 (29.7)	3 (3.7)	12 (48.0)	39 (41.5)	121 (24.0)
**Occupation**							
Formal remunerated	9 (10.7)	21 (16.3)	13 (14.3)	14 (17.3)	6 (24.0)	33 (35.1)	96 (19)
Irregular income^1^	5 (6)	48 (37.2)	13 (14.3)	22 (27.2)	2 (8.0)	10 (10.6)	100 (19.8)
No own income^2^	34 (40.5)	11 (8.5)	15 (16.5)	21 (25.9)	4 (16.0)	14 (14.9)	99 (19.6)
Formal health professional	27 (32.1)	38 (29.5)	31 (34.1)	14 (17.3)	13 (52.0)	32 (34)	155^3^ (30.8)
Informal or traditional health provider	4 (4.8)	9 (7.0)	8 (8.8)	6 (7.4)	0 (0.0)	1 (1.1)	28 (5.6)
Clergy	5 (6.0)	2 (1.6)	11 (12.1)	4 (4.9)	0 (0.0)	4 (4.3)	26 (5.2)
**Religion**							
Christian^4^	72 (85.7)	128 (99.2)	2 (2.2)	56 (69.1)	21 (84)	1 (1.1)	280 (55.6)
Muslim	2 (2.4)	1 (0.8)	89 (97.8)	3 (3.7)	1 (4)	93 (98.9)	189 (37.5)
Animist	8 (9.5)	0 (0.0)	0 (0.0)	21 (25.9)	0 (0.0)	0 (0.0)	29 (5.8)
Atheist	0 (0.0)	0 (0.0)	0 (0.0)	1 (1.2)	1 (4.0)	0 (0.0)	2 (0.4)
Not known	2 (2.4)	0 (0.0)	0 (0.0)	0 (0.0)	2 (8.0)	0 (0.0)	4 (0.8)

Values are *n* (percent) unless indicated otherwise.

^1^Individuals with income from small business, subsistence farming, fishery and livestock, or casual labour.

^2^Individuals who are students, housewives, unemployed, or retired.

^3^ Of note, the number of formal health professionals plus informal/traditional health providers does not equal the total number of individuals in the health providers target group because nine participants from the relatives of deceased people group were also formal health professionals. Moreover, one participant from the health providers target group was a retired community health worker.

^4^Catholic, Protestant or Evangelist, or Christian undetermined.

### Willingness to Know the Cause of Death and Hypothetical Acceptability of Minimally Invasive Autopsies among All Study Participants

Seventy-five percent of the participants (379/504), which included key informants, health providers, and relatives of deceased individuals in all study sites, mentioned that they would be willing to know the CoD of a relative, and an additional 16% (82/504) would be willing to know the CoD only under certain circumstances (see S1 Table). These circumstances included, among others, sudden deaths, unclear clinical diagnoses, or need to resolve witchcraft accusations within the family or the community.

The practice of the MIA on a deceased relative was theoretically acceptable among 73% (366/504) of all participants (Table 3), and a further 14% (69/504) would also accept it conditionally (see S2 Table). The acceptability of the MIA was conditioned on, among others, prior approval by religious and community leaders, cases of sudden death, circumstances where the clinical diagnosis was unclear, circumstances where there was a need to protect the families or the community, or there being no extra costs for the families.

**Table 3:** Willingness to know the cause of death of a relative and hypothetical acceptability of the minimally invasive autopsy for a deceased relative, according to site and interviewed participants’ socio-demographic characteristics.

Characteristic	Willing to Know the Cause of Death		Hypothetical Acceptability of the Minimally Invasive Autopsy	
	*n/N*	Percent	*n/N*	Percent
**By site**				
Lambaréné, Gabon	67/84	79.8	55/84	65.5
Kisumu, Kenya	81/129	62.8	97/129	75.2
Bamako, Mali	82/91	90.1	81/91	89.0
Manhiça, Mozambique	66/81	81.5	63/81	77.8
Maputo, Mozambique	18/25	72.0	19/25	76.0
Karachi, Pakistan	65/94	69.1	51/94	54.3
**By gender**				
Male	240/316	75.9	224/316	70.9
Female	139/188	73.9	142/188	75.5
**By age**				
18–29 y	59/83	71.1	65/83	78.3
30–49 y	180/236	76.3	170/236	72.0
>50 y	140/185	75.7	131/185	70.8
**By education**				
No schooling	37/59	62.7	38/59	64.4
Primary	90/133	67.7	99/133	74.4
Secondary	66/91	72.5	59/91	64.8
Quranic school	20/22	90.9	19/22	86.4
Professional training–health	54/63	85.7	51/63	80.9
Professional training–other	12/15	80.0	10/15	66.6
University or higher–health	65/69	94.2	61/69	88.4
University or higher–other	35/52	67.3	29/52	55.7
**By occupation**				
Regular income	70/96	72.9	64/96	66.7
Irregular income^1^	65/100	65.0	74/100	74.0
No own income^2^	67/99	67.7	63/99	63.6
Formal health professional	135/155	87.1	127/155	81.9
Informal/traditional health provider	21/28	75.0	21/28	75.0
Clergy	21/26	80.8	17/26	65.4
**By religion**				
Christian^3^	208/280	74.3	211/280	75.4
Muslim	148/189	78.3	133/189	70.4
Animist	20/29	69.0	20/29	69.0
Atheist	2/2	100.0	1/2	50.0
Not known	1/4	25.0	1/4	25.0
**Total**	**379/504**	**75.2**	**366/504**	**72.6**

^1^Individuals with income from small business, subsistence farming, fishery and livestock, or casual labour.

^2^Individuals who are students, housewives, unemployed, or retired.

^3^Catholic, Protestant or Evangelist, or Christian undetermined.

When comparing across sites, both the interest in knowing the CoD and the hypothetical acceptability of the MIA were the highest in Mali (90%, 82/91, and 89%, 81/91, respectively) across all target groups, while the willingness to know the CoD was the lowest in Kenya (63%, 81/129), despite the fact that the reported overall hypothetical acceptability of the MIA in this setting was 75% (97/129). In Pakistan, 69% (65/94) of respondents from all target groups were willing to know the CoD, but the acceptability of the MIA was the lowest (54%, 51/94) of all study sites.

By education, participants with a health-related university degree reported the highest willingness to know the CoD and acceptability of the MIA across sites (94.2%, 65/69, for willingness to know the CoD and 88.4%, 61/69, for acceptability of the MIA).

By target group, acceptability across sites was the highest among formal health professionals (87%, 152/175, and 81%, 142/175, for willingness to know the CoD and acceptability of the MIA, respectively). Acceptability among informal and traditional healthcare providers was 75% (21/28) for both willingness to know the CoD and acceptability of the MIA. Among relatives of deceased individuals, 64% (124/194) were willing to know the CoD, and 70% (136/194) hypothetically would accept the MIA. Regarding key informants, 76% (103/135) were willing to know the CoD, and 65% (88/135) would accept the MIA. Among key informants, although the majority of clerics (81%, 21/26) would be willing to know the CoD, a slightly lower proportion (65%, 17/26) would hypothetically accept MIA on a relative.

### Willingness to Know the Cause of Death and Hypothetical Acceptability of the Minimally Invasive Autopsy by Time since Death

Overall, 64% (124/194) of relatives of deceased individuals would be willing to know the CoD, and 70% (136/194) would hypothetically accept the MIA (see S1 and S2 Tables). Of note, when health professionals (nine in total) were excluded from the analysis, we observed no differences in the figures (64.3%, 119/185, for willingness to know the CoD and 70.8%, 131/185, for acceptability of the MIA).

In the overall group of relatives of deceased individuals across all sites, there were no differences in willingness to know the CoD based on the gender or population group (maternal death, stillbirth/newborn death, child death, adult death, elder death) of the deceased. The exception was the relatives of deceased elders (>60 y old), for whom the acceptability was lower (58%, 33/57) than for the other population groups of the deceased. Hypothetical acceptability of the MIA, like willingness to know the CoD, did not differ according to the gender of the deceased. However, there were differences in the acceptability of the MIA according to the population group of the deceased, with slightly higher acceptance for stillbirths and newborn deaths (74%, 17/23), and lower acceptance for pregnant women (65%, 17/26), compared with the other population groups (Table 4).

**Table 4:** Willingness to know the cause of death and hypothetical acceptability of the minimally invasive autopsy among relatives of deceased people, by socio-demographic characteristics of the deceased and kinship relation.

Characteristic	Willing to Know the Cause of Death		Hypothetical Acceptability of the Minimally Invasive Autopsy	
	*n/N*	Percent	*n/N*	Percent
**By time since death**				
0–24 h after death	17/24	70.8	18/24	75.0
1–7 d after death	53/78	67.9	57/78	73.1
30–40 d after death	55/93	59.1	62/93	66.7
**By gender of the deceased**				
Male	64/99	64.6	71/99	71.7
Female	61/96	63.5	66/96	68.8
**By population group of the deceased**				
Stillbirth/newborn death	16/23	69.6	17/23	73.9
Child death (>1 mo–14 y old)	14/21	66.7	15/21	71.4
Adult death (15–60 y old)	44/68	64.7	49/68	72.1
Maternal death (pregnant women)	18/26	69.2	17/26	65.4
Elder death (>60 y old)	33/57	57.9	39/57	68.4
**By religion of the deceased** ^1^				
Christian^2^	68/111	61.2	80/111	72.0
Muslim	43/65	66.1	40/65	61.5
Animist	9/12	75.0	11/12	91.6
Atheist	3/5	60.0	4/5	80.0
Not known	1/2	50.0	1/2	50.0
**By participant kinship relation with the deceased**				
Mother	17/28	60.7	19/28	67.9
Father	8/13	61.5	7/13	53.8
Widow/widower	14/26	53.8	18/26	69.2
Son/daughter	20/31	64.5	22/31	71.0
Sibling	12/18	66.7	11/18	61.1
Grandparent	10/11	90.9	10/11	90.9
Uncle/aunt	13/19	68.4	13/19	68.4
Neighbour/family friend	1/1	100.0	1/1	100.0
Other^3^	27/48	56.3	36/48	75.0

^1^For cases of stillbirth/newborn death and child death, the religion of the parents or legal guardians.

^2^Catholic, Protestant or Evangelist, or Christian undetermined.

^3^Second-grade family: in-law, co-wife, grandchild, cousin, nephew or niece, or godmother.

Regarding the relationship of the respondent with the deceased person, 90% (10/11) of the respondents expressed willingness to know the CoD when the deceased was a grandchild. In contrast, when the deceased was the partner, only 54% (14/26) of the respondents expressed willingness to know the CoD. When the deceased was an offspring, 68% (19/28) of the mothers and 54% (7/13) of the fathers would hypothetically accept the MIA to be performed.

Regarding acceptability of the MIA by religion of the deceased relative, 92% would accept it if the deceased was Animist (11/12), 80% (4/5) if Atheist, 72% (80/111) if Christian, and 61% (40/65) if Muslim.

The willingness to know the CoD was 71% (17/24) for interviews conducted within the first 24 h after the death of a relative, and this proportion diminished with time elapsed after death (68%, 53/78, for relatives interviewed 1–7 d after the death, and 59%, 55/93, for those interviewed 30–40 d after the death). Relatives interviewed within 24 h after the death had higher acceptance of the MIA (75%, 18/24), compared to those interviewed 1–7 d (73%, 57/78) and 30–40 d (67% 62/93) after the death (Table 4).

### Perceived Advantages and Concerns Related to Cause of Death Determination and the Minimally Invasive Autopsy

Box 1 summarises the overall themes that emerged from the analysis of the perceived advantages and concerns expressed by the participants in all target groups, which may constitute facilitators or barriers to the successful implementation of the MIA.

**Box 1. Hypothetical Facilitators and Barriers for the Implementation of the Minimally Invasive Autopsy**

**Hypothetical Facilitators for the Implementation of the MIA**

**Community involvement**

- Support from leaders, health professionals, and media
- The presence of a community witness during sample collection

**Information and transparency**

- Clear information about the procedure and its importance
- Feedback to the families

**Health system requirements**

- Good reputation of the health facility performing MIAs
- Health professional attitudes and preparedness
- Integration of MIAs within the health system

**Traditional post-mortem practices involving invasive procedures**

- Traditional cesarean procedures
- Traditional autopsies

**Incentives/cost reduction**

- Provision of transport
- Costs related to body release, vigil, burial, and related ceremonies
- Body preservation
- Treatment for the family if applicable
- MIA inclusion in health insurance schemes (specifically in Gabon)

**Hypothetical Barriers for the Implementation of the MIA**

**Concerns related to the body/soul**

- Removal of body parts
- Disrespect of the deceased
- Deceased will not rest in peace after MIA

**Religious beliefs**

- Islam and certain Christian sects with religious prohibitions against body mutilation

**Traditional beliefs**

- Still births and neonatal deaths kept secret

**Fear of breach of confidentiality**

- In relation to diseases carrying stigma (e.g., HIV)

**Perceived inappropriateness**

- Inappropriate/unnecessary for certain groups: women, children, elders
- Unnecessary if the clinical diagnosis is clear
- Does not bring the dead back to life

**Misunderstandings regarding the procedure and outcome**

**Reluctance from health professionals**

- Perceived increased workload
- MIAs may reveal clinical misdiagnosis
- Only necessary if diagnosis is inconclusive
- MIA’s margin of error
- Biosafety/risk of infection

**Unpreparedness of health systems**

- Lack of equipment
- Financial limitations
- Competing priorities (services for dead patients versus living ones; MIAs versus existing CoD determination procedures)

**Mistrust**

- Suspicion of new procedures
- Mistrust of the health facility and the providers

**Family issues**

- State of mind around the death
- Complex decision-making processes
- Concerns with timing of ceremonies and burial
- MIA costs for the family

**Access**

- Limitations for identifying deaths at home

**Rumours**

- For example, blood selling

One of the most recurrent perceived advantages of knowing the CoD, regardless of the procedure, was the use of this information for the protection of family members and/or the community from infectious or inheritable diseases. In Mali, where acceptability of the MIA was the highest, concern regarding epidemics such as Ebola was an important factor for hypothetical acceptance. Across sites, knowledge of the CoD was also described as potentially beneficial for avoiding conflicts related to witchcraft accusations and giving peace of mind to the family. Moreover, some participants saw the CoD information as a contribution to science and medical assistance. Formal health professionals in all sites stressed that CoD determination would improve public health policies and would allow them to protect themselves against accusations of malpractice. The latter advantage was also expressed by the informal and traditional healthcare providers.

The above-mentioned perceived advantages of CoD determination also applied to the MIA. Additionally, the nature of the procedure—which was perceived as fast, easy, and requiring only small samples, without leaving visible marks or disfiguring the body—was seen as a critical advantage by most interviewees.

In terms of concerns that may make people not willing to know the CoD, some participants from the three target groups felt that the information obtained would not offer any additional value since the person was already dead. Moreover, some people (specifically, religious leaders and relatives of deceased individuals) considered that death was beyond their control, as reflected in expressions associating death with “God’s will”, “destiny”, or “human nature”. It was also cited that the CoD disclosure could negatively affect the already fragile state of mind of relatives. For instance, in Karachi, it was highlighted that knowing the CoD could lead to remorse if the death was attributed to a curable disease. Fear of breach of confidentiality, especially linked to HIV/AIDS disclosure, was widely mentioned in Manhiça and Kisumu. In Kisumu, there was concern that the traditional practice of wife inheritance within the family of the deceased (i.e., the norm that dictates that the widow remarry a brother or close relative of the deceased) might be jeopardised by the disclosure of the deceased person’s HIV status.

An important concern raised regarding the MIA was the perception that the procedure could come across as a disrespectful, torturing, humiliating, or disturbing act perpetrated on the deceased, especially in some settings, such as Pakistan and Mali, where the belief that the deceased can still feel pain is common. These beliefs were mainly reported among Muslim participants from all target groups. Another barrier mentioned across all sites was that the MIA could interfere with the timing of body release, delaying ceremonies and burial. There was also the perception that it could entail financial burden for families. Moreover, in Gabon, Kenya, and Pakistan, some formal health professionals raised the concern that the MIA results could be used to question the clinician’s pre-mortem diagnosis.

## Discussion

Implementation of any post-mortem procedure (irrespective of its nature) to help refine the estimation of CoD in areas where post-mortem procedures have been seldom utilised requires a profound understanding of what is culturally and religiously acceptable and feasible. Furthermore, understanding how, when and by whom, and in which context grieving relatives of a deceased person should be approached to grant permission to perform such procedures is critical. The objective of this study was to assess the willingness to know the CoD and the hypothetical acceptability of the MIA in six distinct geographical contexts, in preparation for the future integration of such procedures in mortality and CoD surveillance.

This socio-behavioural study is the first to our knowledge to explore the willingness to know the CoD and the hypothetical acceptability of carrying out MIAs on deceased individuals, among a range of different people from low- and middle-income settings from Africa and Asia. While data from previous studies suggested low acceptability for CDAs [2,14,15], we hypothesized that acceptability of the MIA would be higher, and indeed the findings of this study revealed that the majority (over 70%) of the participants interviewed would be willing to know the CoD and would accept the MIA being performed on a relative if this was requested in a potentially real situation. Importantly, this was also the case for individuals who had recently experienced the death of a relative. A crucial finding for the potential implementation of the MIA was the high hypothetical acceptability of the procedure among those who were interviewed within the first 24 h of the death of a relative. Having this target group was an important feature of this study because it is intuitively expected that individuals’ state of mind within the first hours after the death of a relative could negatively affect the acceptability of the MIA. Additionally, it was observed that family members were more likely to accept the MIA within hours after the death compared to weeks after the death. This is in line with the findings of a previous study assessing consent for CDAs [16].

Barriers for the CDA have been identified in previous studies, and they include religious and traditional beliefs [1,2,14,17–19], concerns related to the manipulation of the body (mutilation, disfigurement, organ removal) [1,17,20–22], and delay of the funeral [1,17,19,23], among others. While the barriers identified in this study are consistent with the above-mentioned, those related to mutilation of the body were not present, on account of the minimally invasive nature of the procedure. Importantly, concerns were raised regarding prioritising diagnostic resources for dead individuals—for the more hypothetical goal of preventing future deaths—over living individuals to save the lives. The fear of breaching of confidentiality, particularly related to HIV/AIDS disclosure, was found to be an important barrier in Kenya and Mozambique—both places showing high prevalence of HIV infection [24,25]. The hypothetical acceptability of the performance of the MIA on a relative found in such settings can be jeopardised by concerns regarding the knowledge of the relative’s HIV serostatus, particularly in the case of the spouse.

Formal health professionals were the target group that expressed the highest acceptability of the MIA. Those who would not accept the MIA in their capacity as health professionals raised concerns such as reluctance to approach bereaved families, fears of upsetting or shocking reactions, an increase in their workload, and, importantly, the fear of revealing errors in the pre-mortem diagnosis and management. All of these concerns have been previously reported in studies of the acceptability of the CDA [2,14,16,26–29]. In a study in the UK, healthcare professionals considered the MIA as or more acceptable than the CDA; however, its perceived accuracy was considered to be an important limitation [11]. In the current study, the formal health sector professionals, in addition to the above-mentioned concerns, expressed concerns regarding a potential risk of infection when performing the MIA and the perception that the MIA may be necessary only when the CoD is unclear.

Religious leaders, in particular Muslim leaders, would generally accept the MIA on their relatives only under certain circumstances, such as in the case of sudden death, despite the fact that the majority of them were willing to know the CoD and the fact that the MIA was generally acceptable for them if it was done in other community members. Those barriers specifically related to Islam and reported from Pakistan confirm the findings from the literature regarding the perception that touching, cutting, and injecting are not allowed on a body, since these practices are considered disrespectful or are believed to generate pain to the deceased [10,18,23,30,31]. Additionally, given the religious perspective that death is associated with “God´s will”, seeking to know the CoD was viewed as unnecessary, particularly among (Muslim) religious leaders and relatives of deceased individuals in Mali and Pakistan. Also in the context of Islam, practical concerns such as delays regarding burial ceremonies were also raised. Still, Mali, a predominantly Muslim country, showed high acceptability rates, especially among those with a Quranic school educational background. Contextual factors have to be taken into account when interpreting these results since data collection was undertaken during the Ebola epidemic that affected the country [32], which could have contributed to a generally positive view towards knowing the CoD and thereby helping to prevent the spread of new infections. It is important to highlight that, contrary to preconceptions on this topic, and supporting previous findings, key Islamic authorities such as imams and Quranic masters revealed being willing to accept autopsies and post-mortem procedures when “necessity permitted the forbidden”, particularly in view of their public health benefit [14,23].

In contrast to previous studies reporting parental refusal of post-mortem examinations [15,18,28], a high acceptability of the MIA and willingness to know the CoD among families of cases of stillbirth and neonatal death was observed. This finding was linked to the desire to know the exact CoD and, in agreement with a previous report, to avoid problems in future pregnancies [22]. Acceptance of the MIA for infants and older children was also high. However, the findings from the current study suggest that the interest in knowing the CoD for elders (>60 y old) was lower than for younger people, as seen in other studies [10]. This might be explained by the sense of inevitability of the death of an elder person. The acceptability of the MIA for pregnant women was also low compared to other population groups (65%), possibly attributable to taboos and cultural beliefs related to pregnancy, which are yet to be explored in future studies. This topic has not been sufficiently well documented by existing literature, as studies of community perspectives on maternal deaths in these or similar settings are scarce.

Very few studies have addressed facilitators and barriers for the MIA. A study in Bangladesh found that the MIA was acceptable because it was rapid and non-invasive, minimised burial delays, and could be useful in preventing some diseases affecting the community. Information, community leaders’ support, and the possibility of a witness observing the procedure were mentioned as facilitators [10]. These findings are consistent with the present study, where community involvement, information, transparency, and rapport building of the team were identified as key conditions for the success of potential implementation of the MIA. Furthermore, in this study several forms of incentives and existing post-mortem cultural practices were identified as important facilitators, starting with the perspective that the MIA could be accepted on the condition that it would not pose additional costs for the families.

The major limitation of this study is that the MIA was discussed with the participants as a hypothetical situation. However, the interviews carried out with relatives of deceased people (especially those that were done shortly after the death) are likely to be as close as possible to the real circumstances. While the results of the study are very encouraging in view of a potential use of the MIA for CoD investigation in low-income settings, they remain theoretical and need to be confirmed with the assessment of real-life acceptability during the process of MIA implementation. An additional limitation involves the fact that four of the six study sites were covered by HDSSs. Such populations are typically heavily scrutinized and are also frequently subject to community engagement about the potential benefits of health surveys, and often benefit from research activities. Such a heavy involvement with the populations of the sites where the study took place may have influenced some of the opinions of the participants, possibly towards willingness to participate in this study and also towards a favourable opinion of MIA and CoD determination, in comparison to what would have occurred in settings with less surveillance activity and less community involvement in addressing health issues. Finally, the MIA tool may perform differently in different settings, according to the underlying epidemiology of life-threatening conditions. In settings where infectious diseases are the predominant cause of mortality, such as the ones where this study was conducted, it is expected that the MIA would deliver reliable results, thus providing the necessary data to inform the family of the deceased. However, in settings where the main drivers of mortality are non-communicable (i.e., high-income countries), MIA may not be able to robustly reach a conclusive diagnosis, and this could end up leading to underperformance of the tool and eventual lower acceptability at the community level.

### Conclusions

This study has shown that the MIA may be acceptable in places where post-mortem procedures were hitherto believed to be unfeasible. Gathering socio-anthropological information is critical prior to any future implementation of MIA procedures, and will allow locally tailored recommendations to be put in place beforehand and throughout the implementation process. Early community engagement and transparency in terms of the information provided to family and community members are key for optimising community acceptability. Additionally, health systems should be prepared to ensure a prompt response following a death, addressing logistical, human, and material resources, and guaranteeing the necessary sensibility and human rapport from health professionals asking for consent for and performing the MIA. In view of the promising role of the MIA in low- and middle-income countries, this study contributes to the scarce body of knowledge about barriers and facilitators for implementing minimally invasive post-mortem diagnostic procedures.

## Supporting Information

S1 Checklist COREQ checklist for the study. (PDF)

S1 Table Willingness to know the cause of death of a relative, according to site, interviewed participants’ socio-demographic characteristics, and target group. (PDF)

S2 Table Hypothetical acceptability of the minimally invasive autopsy for a deceased relative, according to site, interviewed participants’ socio-demographic characteristics, and target group. (PDF)

S1 Text Protocol describing procedures, methodology, and analysis plan used during the study. (PDF)

## Abbreviations

CDA: complete diagnostic autopsy
CoD: cause of death
HDSS: Health and Demographic Surveillance System
MIA: minimally invasive autopsy
