# Supplementary material for: Willingness to Know the Cause of Death and Hypothetical Acceptability of the Minimally Invasive Autopsy in Six Diverse African and Asian Settings: A Mixed Methods Socio-Behavioural Study
Source: PLoS Med. 2016 Nov 22;13(11):e1002172. doi: 10.1371/journal.pmed.1002172 (PMC5119724; doi:10.1371/journal.pmed.1002172)
Supplement: S1 Checklist — (PDF) [file pmed.1002172.s001.pdf]

Consolidated criteria for reporting qualitative studies (COREQ): 32-item checklist

Manuscript: Willingness to Know the Cause of Death and Hypothetical Acceptability of the Minimally Invasive Autopsy in Five Diverse African and Asian Settings: A Mixed Methods Socio-behavioural Study

Date: August, 16th 2016

DOMAIN 1: RESEARCH TEAM AND REFLEXIVITY

Personal characteristics

1. **Interviewer/facilitator: Which author/s conducted the interview or focus group?**

   Maria Maixenchs, Rui Anselmo, Clarah Akello, Maureen Ondire, Shujaat H. Zaidi, Sajid Bashir Soofi, Kounandji Diarra, Mahamane Djiteye and Pamela Cathérine Angoissa Minsoko.

2. **Credentials:**
   Researcher`s credentials include PhD, Msc and Bsc.

3. **Occupation:**
   Researcher's occupations at the time of the study include social scientists, researchers, research assistants, site study coordinators, PIs and research professors.

4. **Gender:** Eight of the researchers are women and 13 are men.

5. **Experience and training**
   All researchers had a minimum of 3 years´ experience on global health, social sciences and/or qualitative research.

Relationship with participants

6. **Relationship established**
   The interviewers did not have an ongoing relationship with the interviewees.

7. **Participant knowledge of the interviewer**
   The context of the interviews and the project overall was informally introduced when interviewers arrived at the home/office of the interviewee, and formally as part of the informed consent process.

8. **Interviewer characteristics:** Data were collected by social scientists and research assistants.

DOMAIN 2: STUDY DESIGN

Theoretical framework

9. **Methodological orientation and Theory:** Content analysis and thematic analysis.

Participant selection

10. **Sampling**: Participants were by convenience and purposely selected.

11. **Method of approach.** Participants were approached face-face at the health facilities, at their residences, at their workplace or at community meetings.

12. **Sample size:** 504 participants were included in the study.

13. **Non-participation:**

    Twenty-eight people refused to participate. Reasons for non-participation were mainly related to the state of mind around the death which was incompatible with being interviewed (15 persons), being 4 of them too resentful to talk about their experience. Six persons refused to talk because they had no time, 2 because they had to travel, 2 asked for money in exchange for their time and 3 people did not give any reason.

Setting

14. **Setting of data collection:** Data were collected at the health facilities, residences and workplaces of participants.

15. **Presence of non-participants:** A few interviews were done in the presence of non-participants, and only under request of the participant

16. **Description of sample** There were 3 target groups:

    Key informants

    a) Someone who has the privilege to know the community, and/ or can influence the opinion of the community regarding the phenomenon of "death"

    b) Someone who knows the rituals and ethnic/ religious norms and requirements for death-related events

    Health providers:

    a) A health care provider who is regularly in contact with death, specifically at the time of death

    b) Professionals in regular contact with death

Relatives of deceased people are defined as the closest possible relative to the deceased, not necessary legally related to the deceased, and with decision making power on family health and death issues, i.e. somebody naturally appointed by the family.

a) Those who have suffered a death between 30-40 days earlier
b) Those who have suffered a death between 1-7 days earlier
c) Those who have suffered a death between 0-24 hours earlier

Data collection

17. **Interview guide: Were questions, prompts, guides provided by the authors?** Yes. **Was it pilot tested?** Yes.

18. **Repeat interviews: Were repeat interviews carried out?** No.

19. **Audio/visual recording:** Audio recording were used to collect the data.

20. **Field notes:** Field notes were made when collecting the data.

21. **Duration:** Duration of the interviews were variable. The duration of the interviews was around 1 hour - 1 hour and a half, but those of relatives who suffered a death within 24 hours were around 40 minutes.

22. **Data saturation:** Data saturation was discussed.

23. **Transcripts returned:** Transcripts were not returned to participants for comments or corrections.

DOMAIN 3: ANALYSIS AND FINDINGS

Data analysis

24. **Number of data coders:** N/A. Framework analysis was used.

25. **Description of the coding tree:** N/A

26. **Derivation of themes:** Themes identified were derived from the data.

27. **Software:** Excel spreadsheets.

28. **Participant checking:** Participants did not provide feed-back on the findings.

Reporting

29. **Quotations presented: N/A**

30. **Data and findings consistent:** There was consistency between the data presented and the findings.

31. **Clarity of major themes:** The major themes were clearly presented in the findings.

32. **Clarity of minor themes:** There is no description of diverse cases or discussion of minor themes.
