# Supplementary material for: Willingness to Know the Cause of Death and Hypothetical Acceptability of the Minimally Invasive Autopsy in Six Diverse African and Asian Settings: A Mixed Methods Socio-Behavioural Study
Source: PLoS Med. 2016 Nov 22;13(11):e1002172. doi: 10.1371/journal.pmed.1002172 (PMC5119724; doi:10.1371/journal.pmed.1002172)
Supplement: S1 Table — (PDF) [file pmed.1002172.s002.pdf]

S1 Table

Willingness to know the cause of death of a relative, according to site, target group, and interviewed participants' socio-demographic characteristics.

	Yes	Under certain circumstances	No	Do not know	Did not answer
	Percent (n/N)	Percent (n/N)	Percent (n/N)	Percent (n/N)	Percent (n/N)
**By site**					
**Lambaréné, Gabon**	79.8 (67/84)	14.3 (12/84)	4.8 (4/84)	0.0 (0/84)	1.2 (1/84)
**Kisumu, Kenya**	62.8 (81/129)	30.2 (39/129)	1.6 (2/129)	0.8 (1/129)	4.7 (6/129)
**Bamako, Mali**	90.1 (82/91)	3.3 (3/91)	2.2 (2/91)	4.4 (4/91)	0.0 (0/91)
**Manhiça, Mozambique**	81.5 (66/81)	1.2 (1/81)	9.9 (8/81)	2.5 (2/81)	4.9 (4/81)
**Maputo, Mozambique**	72.0 (18/25)	12.0 (3/25)	8.0 (2/25)	0.0 (0/25)	8.0 (2/25)
**Karachi, Pakistan**	69.1 (65/94)	25.5 (24/94)	5.3 (5/94)	0.0 (0/94)	0.0 (0/94)
**TOTAL**	75.2 (379/504)	16.3 (82/504)	4.6 (23/504)	1.4 (7/504)	2.6 (13/504)
**By target group**					
**Key Informants**	76.3 (103/135)	14.8 (20/135)	5.9 (8/135)	0.0 (0/135)	3.0(4/135)
**Health providers**	86.9 (152/175)	7.4 (13/175)	2.9 (5/175)	0.6 (1/175)	2.3 (4/175)
**Relatives of deceased people**	63.9 (124/194)	25.3 (49/194)	5.2 (10/194)	3.1 (6/194)	2.6 (5/194)
**By gender**					
**Male**	75.9 (240/316)	16.5 (52/316)	4.1 (13/316)	0.6 (2/316)	2.8 (9/316)
**Female**	73.9 (139/188)	16.0 (30/188)	5.3 (10/188)	2.7 (5/188)	2.1 (4/188)
**By age**					
**18-29 y**	71.1 (59/83)	18.1 (15/83)	7.2 (6/83)	1.2 (1/83)	2.4 (2/83)
**30-49 y**	76.3 (180/236)	17.4 (41/236)	3.0 (7/236)	0.8 (2/236)	2.5 (6/236)
**>50 y**	75.7 (140/185)	14.1 (26/185)	5.4 (10/185)	2.2 (4/185)	2.7 (5/185)

**By education**					
**No schooling**	62.7 (37/59)	22.0 (13/59)	10.2 (6/59)	5.1 (3/59)	0.0 (0/59)
**Primary**	67.7 (90/133)	18.8 (25/133)	4.5 (6/133)	1.5 (2/133)	7.5 (10/133)
**Secondary**	72.5 (66/91)	24.2 (22/91)	3.3 (3/91)	0.0 (0/91)	0.0 (0/91)
**Quranic School**	90.9 (20/22)	0.0 (0/22)	0.0 (0/22)	9.1 (2/22)	0.0 (0/22)
**Professional Training – Health**	85.7 (54/63)	9.5 (6/63)	4.7 (3/63)	0.0 (0/63)	0.0 (0/63)
**Professional Training - Other**	80.0 (12/15)	13.3 (2/15)	6.6 (1/15)	0.0 (0/15)	0.0 (0/15)
**University or higher – Health**	94.2 (65/69)	0.0 (0/69)	1.4 (1/69)	0.0 (0/69)	4.3 (3/69)
**University or higher - Other**	67.3 (35/52)	27.9 (14/52)	5.7 (3/52)	0.0 (0/52)	0.0 (0/52)
**By occupation**					
**Regular income**	72.9 (70/96)	19.8 (19/96)	5.2 (5/96)	0.0 (0/96)	2.1 (2/96)
**Irregular income**[1]	65.0 (65/100)	28.0 (28/100)	3.0 (3/100)	2.0 (2/100)	2.0 (2/100)
**No own income**[2]	67.7 (67/99)	17.2 (17/99)	8.1 (8/99)	3.0 (3/99)	4.0 (4/99)
**Formal health professional**	87.1 (135/155)	7.7 (12/155)	2.6 (4/155)	0.0 (0/155)	2.6 (4/155)
**Informal/Traditional health provider**	75.0 (21/28)	14.3 (4/28)	7.1 (2/28)	3.6 (1/28)	0.0 (0/28)
**Clergy**	80.8 (21/26)	7.7 (2/26)	3.8 (1/26)	3.8 (1/26)	3.8 (1/26)
**By religion**					
**Christian**[3]	74.3 (208/280)	17.5 (49/280)	3.6 (10/280)	0.7 (2/280)	3.9 (11/280)
**Muslim**	78.3 (148/189)	14.3 (27/189)	5.3 (10/189)	2.1 (4/189)	0.0 (0/189)
**Animist**	69.0 (20/29)	10.3 (3/29)	10.3 (3/29)	3.4(1/29)	6.9 (2/29)
**Atheist**	100.0 (2/2)	0.0 (0/2)	0.0 (0/2)	0.0 (0/2)	0.0 (0/2)
**Not known**	25.0 (1/4)	75.0 (3/4)	0.0 (0/4)	0.0 (0/4)	0.0 (0/4)

[1]Individuals with income from small business, subsistence farming, fishery and livestock, or casual labour.
[2]Individuals who are students, housewives, unemployed, or retired.
[3]Catholic, Protestant or Evangelist, or Christian undetermined.
