# Supplementary material for: Willingness to Know the Cause of Death and Hypothetical Acceptability of the Minimally Invasive Autopsy in Six Diverse African and Asian Settings: A Mixed Methods Socio-Behavioural Study
Source: PLoS Med. 2016 Nov 22;13(11):e1002172. doi: 10.1371/journal.pmed.1002172 (PMC5119724; doi:10.1371/journal.pmed.1002172)
Supplement: S2 Table — (PDF) [file pmed.1002172.s003.pdf]

S2 Table

Hypothetical acceptability of the minimally invasive autopsy for a deceased relative, according to site, target group, and interviewed participants' socio-demographic characteristics.

	Yes	Under certain circumstances	No	Do not know	Did not answer	Not asked
	Percent (n/N)	Percent (n/N)	Percent (n/N)	Percent (n/N)	Percent (n/N)	Percent (n/N)
**By site**						
**Lambaréné, Gabon**	65.5 (55/84)	23.8 (20/84)	9.5 (8/84)	1.2 (1/84)	0.0 (0/84)	0.0 (0/84)
**Kisumu, Kenya**	75.2 (97/129)	6.2 (8/129)	8.5 (11/129)	0.8 (1/129)	0.8 (1/129)	8.5 (11/129)
**Bamako, Mali**	89.0 (81/91)	5.5 (5/91)	2.2 (2/91)	3.3 (3/91)	0.0 (0/91)	0.0 (0/91)
**Manhiça, Mozambique**	77.8 (63/81)	6.2 (5/81)	7.4 (6/81)	3.7 (3/81)	4.9 (4/81)	0.0 (0/81)
**Maputo, Mozambique**	76.0 (19/25)	16.0 (4/25)	0.0 (0/25)	0.0 (0/25)	8.0 (2/25)	0.0 (0/25)
**Karachi, Pakistan**	54.3 (51/94)	28.7 (27/94)	14.9 (14/94)	2.1 (2/94)	0.0 (0/94)	0.0 (0/94)
**TOTAL**	72.6 (366/504)	13.7 (69/504)	8.1 (41/504)	2.0 (10/505)	1.4 (7/504)	2.2 (11/504)
**By target group**						
**Key Informants**	65.2 (88/135)	16.3 (22/135)	8.9 (12/135)	1.5 (2/135)	1.5 (2/135)	6.7 (9/135)
**Health providers**	81.1 (142/175)	10.3 (18/175)	4.6 (8/175)	1.1 (2/175)	1.7 (3/175)	1.1 (2/175)
**Relatives of deceased people**	70.1 (136/194)	14.9 (29/194)	10.8 (21/194)	3.1 (6/194)	1.0 (2/194)	0.0 (0/194)
**By gender**						
**Male**	70.9 (224/316)	15.5 (49/316)	7.3 (23/316)	1.3 (4/316)	1.6 (5/316)	3.5 (11/316)
**Female**	75.5 (142/188)	10.6 (20/188)	9.6 (18/188)	3.2 (6/188)	1.1 (2/188)	0.0 (0/188)
**By age**						
**18-29 y**	78.3 (65/83)	13.3 (11/83)	7.2 (6/83)	0.0 (0/83)	1.2 (1/83)	0.0 (0/83)
**30-49 y**	72.0 (170/236)	15.3 (36/236)	7.2 (17/236)	1.7 (4/236)	1.3 (3/236)	2.5 (6/236)
**>50 y**	70.8 (131/185)	11.9 (22/185)	9.7 (18/185)	3.2 (6/185)	1.6 (3/185)	2.7 (5/185)

**By education**						
**No schooling**	64.4 (38/59)	10.2 (6/59)	18.6 (11/59)	3.4 (2/59)	3.4 (2/59)	0.0 (0/59)
**Primary**	74.4 (99/133)	6.8 (9/133)	9.0 (12/133)	3.8 (5/133)	2.3 (3/133)	3.8 (5/133)
**Secondary**	64.8 (59/91)	25.3 (23/91)	6.6 (6/91)	1.1 (1/91)	0.0 (0/91)	2.2 (2/91)
**Quranic School**	86.4 (19/22)	9.1 (2/22)	0.0 (0/22)	4.5 (1/22)	0.0 (0/22)	0.0 (0/22)
**Professional Training – Health**	80.9 (51/63)	11.1 (7/63)	7.9 (5/63)	0.0 (0/63)	0.0 (0/63)	0.0 (0/63)
**Professional Training – Other**	66.6 (10/15)	13.3 (2/15)	6.6 (1/15)	0.0 (0/15)	0.0 (0/15)	13.3 (2/15)
**University or higher - Health**	88.4 (61/69)	5.8 (4/69)	2.8 (2/69)	0.0 (0/69)	2.8 (2/69)	0.0 (0/69)
**University or higher - Other**	55.7 (29/52)	30.7 (16/52)	7.7 (4/52)	2.0 (1/52)	0.0 (0/52)	2.0 (1/52)
**By occupation**						
**Regular income**	66.7 (64/96)	18.8 (18/96)	6.3 (6/96)	1.0 (1/96)	2.1 (2/96)	5.2 (5/96)
**Irregular income**[1]	74.0 (74/100)	11.0 (11/100)	8.0 (8/100)	3.0 (3/100)	1.0 (1/100)	3.0 (3/100)
**No own income**[2]	63.6 (63/99)	17.2 (17/99)	15.2 (15/99)	3.0 (3/99)	1.0 (1/99)	0.0 (0/99)
**Formal health professional**	81.9 (127/155)	10.3 (16/155)	4.5 (7/155)	0.6 (1/155)	1.9 (3/155)	0.6 (1/155)
**Informal/Traditional health provider**	75.0 (21/28)	10.7 (3/28)	7.1 (2/28)	3.6 (1/28)	0.0 (0/28)	3.6 (1/28)
**Clergy**	65.4 (17/26)	15.4 (4/26)	11.5 (3/26)	3.8 (1/26)	0.0 (0/26)	3.8 (1/26)
**By religion**						
**Christian**[3]	75.4 (211/280)	10.4 (29/280)	6.8 (19/280)	1.8 (5/280)	1.8 (5/280)	3.9 (11/280)
**Muslim**	70.4 (133/189)	17.5 (33/189)	9.5 (18/189)	2.6 (5/189)	0.0 (0/189)	0.0 (0/189)
**Animist**	69.0 (20/29)	13.8 (4/29)	13.8 (4/29)	0.0 (0/29)	3.4 (1/29)	0.0 (0/29)
**Atheist**	50.0 (1/2)	0.0 (0/2)	0.0 (0/2)	0.0 (0/2)	50.0 (1/2)	0.0 (0/2)
**Not known**	25.0 (1/4)	75.0 (3/4)	0.0 (0/4)	0.0 (0/4)	0.0 (0/4)	0.0 (0/4)

[1]Individuals with income from small business, subsistence farming, fishery and livestock, or casual labour.
[2]Individuals who are students; housewives, unemployed, or retired
[3]Catholic, Protestant or Evangelist, or Christian undetermined.
