# Supplementary material for: Willingness to Know the Cause of Death and Hypothetical Acceptability of the Minimally Invasive Autopsy in Six Diverse African and Asian Settings: A Mixed Methods Socio-Behavioural Study
Source: PLoS Med. 2016 Nov 22;13(11):e1002172. doi: 10.1371/journal.pmed.1002172 (PMC5119724; doi:10.1371/journal.pmed.1002172)
Supplement: S1 Text — (PDF) [file pmed.1002172.s004.pdf]

Validation of the Minimally Invasive Autopsy tool for cause of death investigation in developing countries

CaDMIA – Cause of Death using Minimally Invasive Autopsies

FEASIBILITY AND ACCEPTABILITY STUDY
Date: v01_12.07.2013

CaDMIA participating institutions:

- Centro de Investigação em Saúde da Manhiça (CISM), Manhiça, Mozambique
- Hospital Central de Maputo (HCM), Maputo, Mozambique
- Centre de Recherches Médicales de Lambaréné, Albert Schweitzer Hospital (CERMEL, ASH) Lambaréné, Gabon
- CDC/Kenya Medical Research Institute (KEMRI/CDC Research and Public Health Collaboration), Kisumu, Kenya
- Centre pour le Développement de Vaccins in Mali (CVD-Mali), Bamako, Mali
- Department of Pediatrics and Child Health, Aga Khan University (AKU), Karachi, Pakistan
- Barcelona Centre for International Health Research (CRESIB), Hospital Clínic-University of Barcelona, Barcelona, Spain

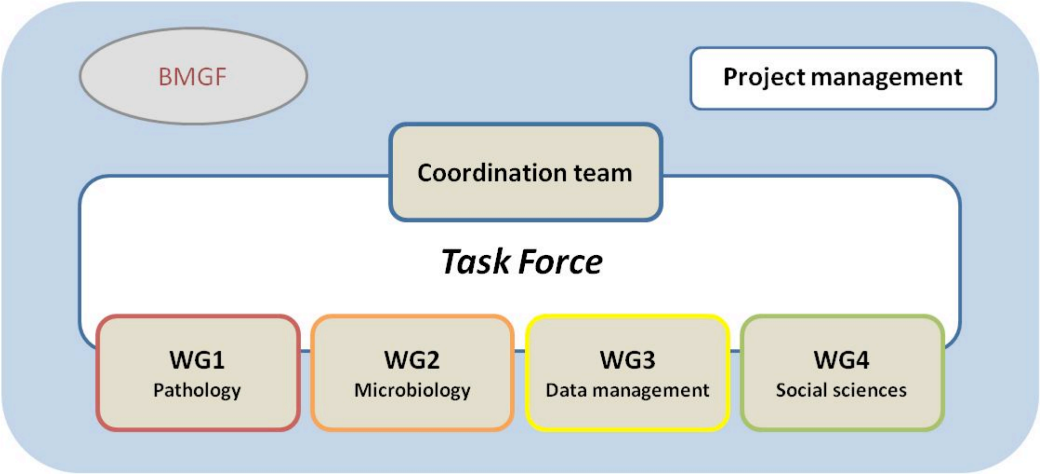

Investigators involved in the Feasibility study (WG4):

- CISM: Khátia Munguambe, Rui Anselmo, Lina Fiosse, Eusébio Macete
- HCM: Carla Carrilho, Mamudo Ismail
- CERMEL, ASH: Selidji T Agnandji, Bertrand Lell
- KEMRI/CDC: Emily Zielinski-Gutiérrez, Frank Odhiambo
- CVD-Mali: Kounandji Diarra, Mahamane Djiteye, Samba Sow
- AKU: Shujaat Zaidi, Zulfiqar Bhutta
- CRESIB: Maria Maixenchs, Quique Bassat, Clara Menéndez

Contents

1. Summary ... 3

2. Background ... 4

3. Justification for a feasibility and acceptability study: ... 5

4. Objectives: ... 5

5. Study Design ... 6

6. Study Sites and Population ... 6

6.1 Study sites ... 6

6.2 Target Population ... 8

6.2.1 Key Informants ... 8

6.2.2 Next of kin of the deceased ... 9

6.2.3 Health systems professionals ... 9

7. METHODOLOGY ... 9

7.1 Recruitment strategy ... 9

7.1.1 Rapport building with the community ... 9

7.1.2 Enrolment ... 9

7.2 Sampling ... 10

7.3 Data collection approaches ... 10

7.3.1 Community-Based Data Collection ... 10

7.3.2 Hospital-Based Data Collection: ... 11

8. Analysis ... 12

9. Ethical Considerations ... 12

10. References ... 13

Annexes:

Annex 1: Timeline

Annex 2: Budget

Annex 3: Informed Consent

Annex 4: Data collection guides

1. Summary

The global health community has not yet adequately resolved how to estimate with precision the most important causes of death. While for developed countries death registries and/or pathological autopsy procedures generally provide valuable information, for the developing world very little evidence- based information is available on cause-specific mortality rates. In such countries, where the majority of deaths occur outside the health facilities, and routine autopsies are not performed, cause of death investigation is often restricted to the practice of verbal autopsies (VA), a method with important limitations. This uncertainty has led to important debates and regarding recent estimates of cause-specific mortality and burden of disease, particularly concerning the contribution of infectious diseases to mortality, which are believed to account for the highest burden in developing settings.

Complete diagnostic autopsies (CDA), the current gold standard methodology to inform on cause of death, may be challenging in rural areas of the developing world, not only due to the lack of technical expertise, but also because they may raise issues contributing to low cultural and/or religious acceptance. Thus, there is an urgent need to validate newer, more acceptable, less invasive approaches that could substitute CDA and complement current VA methods without compromising pathological and microbiological diagnosis. In recent years**, minimally invasive autopsy (MIA) techniques** have been developed with this precise aim. MIA, as it currently stands, includes the use of imaging techniques coupled with the performance of targeted small diagnostic biopsies of key organs. Such techniques produce reliable and comparable results to the CDA. Moreover, in developing settings, MIA could still be performed guided by low-cost ultrasound or even without the imaging guidance, based exclusively in the performance of targeted key organ biopsies.

The confirmation that MIA is an acceptable, feasible, valid and reliable tool to inform on the cause of death in all age-groups would be a major public health achievement, as it would allow the introduction of such simplified techniques as an alternative to CDAs to complement clinical diagnosis and VA. It would also allow a more robust surveillance of the prevalence and trends of those infectious diseases with major mortality burden, and consequently, improved health planning and more targeted prioritization of available resources.

Implementing an invasive (albeit minimally) tool that can help refine the diagnosis of Cause of Death (CoD) provided by non-invasive methods such as the VA, requires a profound understanding of what is culturally and/or religiously acceptable and feasible, and how, when, in which contexts and by whom should grieving relatives of a deceased be approached to grant permission to perform MIAs. Fear of body mutilation, impossibility to obtain consent from the deceased, cultural or religious norms, young age of the deceased, little use to the deceased, and delays in the funeral have previously been identified as key factors underlying refusal for autopsies in general.

We aim **to assess the feasibility and acceptability of using MIA approaches in different cultural, religious and geographical backgrounds.** Such studies should help to understand local attitudes, beliefs and practices related to deaths occurring within or outside the health system, and would be conducted in rural or peri-urban areas of 5 countries (Mozambique, Mali, Kenya, Gabon and Pakistan) in preparation for an eventual larger multicenter MIA study. Such sites have been carefully chosen so as to capture a wide variety of cultural, religious, geographical and epidemiological backgrounds.

This study will use ethnography (a type of anthropological study design) to investigate the local meaning of “death” as a phenomenon from the perspective of the local communities. Data will generated through interviews, observations and focus group discussions involving community key informants, relatives of the deceased, and health professionals. Data analysis will be performed using Nvivo.

2. Background

Historically, the practice of autopsies to deceased individuals has been and remains to date a critical component of the understanding of disease mechanisms and the improvement of medicine. CDA involves the anatomo-pathological examination of the corpse of a deceased person in order to establish the most plausible CoD. It is ideally performed in a laboratory setting with two trained individuals (a medical doctor trained in anatomic pathology and an assistant) and combines an assortment of dissection, macroscopic evaluation, microscopy, microbiology, toxicology and other special techniques, in addition to the deceased's medical history and the evaluation of the circumstances surrounding the death. Indeed, CDAs are still considered the gold standard methodology to establish the CoD in any age group[i], and are used routinely in the developed world in cases where CoD has not been correctly identified. This proposal would not exist if this practice could be directly translated to the developing world, where routine performance of CDA simply does not occur, and is restricted to forensic obligations, to a few tertiary hospitals in the capitals, or to specific research exercises. Reasons for this include, among others, the large proportion of deaths occurring outside the health system, the lack of technical expertise, sufficient equipment, capacity and trained human resources; and also potentially to a strong cultural and/or religious apprehension about the practice of post-mortem procedures from the community perspective.

In addition to the limitations for their use in resource-constrained settings, CDAs have also other important drawbacks and potential limitations worth mentioning. Health professionals are the responsible for the transmission of official information and requests about health and death to the next of kin. Their awareness about autopsy process, relevance and benefits, as well as their skills and attitudes towards approaching a family grieving the loss of a member would highly influence on the acceptance of MIA[ii,iii,iv,v]. Unpredictable and generally low patterns of consent may imply that pathology is only studied in determined places or in a proportion of fatal cases (rather than in all), which may introduce bias if a particular pathogen is common in a group where autopsy was unacceptable for religious or cultural reasons.

In spite of all these limitations, CDAs still remain the gold-standard methodology for CoD determination. The high rate of clinico-pathologic discrepancies in different studies conducted in the developing world (for example in autopsy studies of maternal deaths in Mozambique[vi], or of paediatric deaths in Malawi[vii]) emphasizes the necessary role in the developing world for CDA to ascertain the real CoD, and the limitations of current VA methods. Given that CDA can only be carried out infrequently in poor resource settings, there is an urgent need to validate newer approaches that could substitute or simplify CDA using a culturally acceptable, less invasive and targeted pathological sampling methodology so as to complement currently suboptimal methods without compromising pathological and microbiological diagnoses.

Despite their huge diagnostic value, performing CDAs in developing settings is challenging mainly due to the lack of resources and technical expertise to perform them[viii]. In addition, in such settings, consent rates for CDAs have traditionally been very poor. For example, only a quarter of the families approached in Lusaka consented to an open autopsy following death of their child[ix], and studies in Malawi have shown similar rates, even after a more intense contact with families during their child's illness. In spare studies, consent rates are high but at the cost of high investment in human resources and incentives[1]. Moreover, many of the deaths in such settings occur at the community level, as access to health facilities remains a daily challenge.

Understanding that the long-term feasibility of routinely performing CDAs is problematic, there is an urgent need to develop simpler and feasible methods to ascertain the most accurate CoD, particularly due to the limitations already mentioned related to VA methodologies or clinical diagnosis.

The constant decline of consent rates for autopsy in western countries has stimulated in recent years the development of Minimally Invasive Autopsy (MIA) as an alternative to classic

---

[1] *Experiences in Implementing Verbal Autopsy And Postmortem study For Measuring Tuberculosis Mortality In Kenya.* Peter Nyamthimba Onyango, Gacheri S, Laserson K, Agaya J, Cain K, Sitienei J , Odhiambo F. KEMRI/CDC Research and Public Health collaboration; Division of Leprosy Tuberculosis and Lung Disease; Jomo Kenyatta University of Agriculture and Technology. Data to be published.

pathological autopsy. MIA, as it currently stands, includes the use of imaging techniques coupled with the performance of targeted small diagnostic biopsies (by needle puncture) of key organs. Such techniques have been shown to produce reliable and comparable results to the CDA[x,xi,xii,xiii,xiv] in the developed world, although there is still little experience in their practice.

Even though imaging techniques may not be available in most developing settings, MIA could theoretically still be performed in the absence of imaging guidance, based exclusively in the performance of targeted key organ biopsies. The use of portable, low-cost ultrasound machines may improve the results of blind biopsy[xv].

Understanding the burden of infectious diseases as causes of mortality, even at the expense of potentially underestimating non-infectious causes of death, is particularly relevant because interventions exist with a proven potential to prevent their transmission or limit their consequences using antimicrobial-tailored treatments in the patient.

The confirmation that MIA is a feasible, valid and reliable tool to inform on the CoD could allow the introduction of such simplified techniques as an alternative to CDAs to complement clinical diagnosis and help survey the prevalence of certain infectious diseases important as causes of mortality, proving of great public health utility.

3. Justification for a feasibility and acceptability study

Implementing an invasive (albeit minimally) tool that can help refine the diagnosis of CoD provided by non-invasive methods such as the VA, requires a profound understanding of what is culturally and/or religiously acceptable and feasible, and how, when, in which contexts and by whom should grieving relatives of a deceased be approached to grant permission to perform MIAs. Fear of body mutilation, impossibility to obtain consent from the deceased, cultural or religious norms, young age of the deceased, little use of the information gained to the deceased, and delays in the funeral/burial have previously been identified as key factors underlying refusal for autopsies in general. Further in-depth assessments are needed to verify to what extent these and other, not yet identified factors, are important determinants of MIA acceptability.

4. Objectives

The main purpose of the overall CaDMIA project is to design and assess the performance of MIA tools for the investigation of infectious causes of death, and to evaluate the acceptability and feasibility of using such tools in different cultural, social, religious and geographical backgrounds.

Overall objective of the feasibility and acceptability study:

- To conduct site-specific research to understand local attitudes and perceptions related to death at the community level and the feasibility and acceptability of conducting MIAs in deaths occurring both within and outside the health system.

Specific objectives:

- To document cultural, social and religious norms and conduct around deaths
- To evaluate the willingness to know the cause of death and its implications in different contexts
- To examine community and relative’s attitudes and behaviours towards MIAS in all age groups
- To determine the acceptability of MIA by the relatives of the deceased and community in general
- To identify factors motivating the acceptance and refusal to perform MIA

5. Study Design

The CaDMIA feasibility and acceptability study will be based on sociological and anthropological research approaches. The core approach will be ethnography, which is an iterative, cumulative process whereby the researcher or the research team interact directly with the members of the local communities in their own day-to-day environment, which will allow first to explore the phenomenon (in this case: "death") in its broader sense and to understand its local meaning, before looking more in-depth into the specific study research questions (in this case: acceptability of MIAs).

Generally, the ethnographic research process starting from a number of general topics, about which information is collected through open-ended questions during in-depth interviews and focus group discussions, whereby participants are encouraged to discuss about the topics informally. New questions and probing during the interview, and the topics discussed during subsequent interviews are adjusted depending on what emerges from these discussions based on the original IDIs and FGD's Guidelines. This process continues until a point of saturation is reached – i.e. when no more new information emerges from the data, and no new insights are generated from the discussions. This approach requires flexibility, and as a result no lists of predefined questions are used (as in a questionnaire) but rather a set of broad topics. Observations, reflexive field notes and informal conversations often complete the information collected. Ethnography generates qualitative data, which is analysed through the grounded theory approach (theoretical generalisations emerge from the data rather than being assumed beforehand). Following are the basic 7 characteristics of ethnographic research[2]:

1. Is conducted in natural settings
2. Is done within the field site
3. Provides holistic and systematic overview of the context
4. Documents native perspectives
5. Is descriptive and interpretative
6. Is guided by general research questions not hypothesis
7. Focuses on meaning of word and images rather than in numbers

6. Study Sites and Population

6.1 Study sites

The study will be carried out mainly in rural/semi-rural areas of 4 countries: Manhiça (Mozambique), Lambaréné (Gabon), Karachi (Pakistan) and Kisumu (Kenya). These settings do not conduct autopsies as a routine. Additionally, in Mozambique the study will be conducted in Maputo, an urban setting where CDAs are performed.

Gabon: In Gabon, the study will be conducted in the district of *Ogooué et Lacs* (250 km from Libreville). It is a semi-rural area, with an area of 3700 Km^2 and a population of 25,000 inhabitants. The population's main occupation is agriculture and fishing and the literacy levels are estimated at 85%.

In this study area a DSS covering approximately 28,000 individuals has been established in 2010 with planned annual updates. This area encloses the Albert Schweitzer District Hospital,

[2] Source: Saad Aqeel , R. Campbell; Design Research Brief, IDUS 215 Contextual Research Methods 2012

the Lambaréné Regional District Hospital, the Fougamou Regional Rural Hospital, the Makouke Health care centre and 10 dispensaries, all previously and/or currently involved in clinical research. Several ethnic groups cohabit together in Lambaréné. The dominant and historical groups are Omyene, Fangs, Guischira, Tsogo. Additionaly, several communities of foreigners from other African countries are resident in Lambaréné. Gabon is a laic country where Animism, Christian groups and Islam are the main religions. The socio-economic backgrounds include a majority of resource limited groups and a small middle class.

Kenya: In Kenya, the study will be conducted in the county of Siaya, located in western Kenya near Lake Victoria, located approximately 50-100km from Kisumu, which is the third largest city in Kenya. This is a largely rural area of ~700km^2 with subsistence farming, fishing and small trade comprising the livelihood of most people. The area is endemic for malaria and has roughly doubled the national prevalence of HIV at over 14% (and significantly higher within some communities in this area.) The population is almost entirely of the Luo ethnic group, a community that practices polygamy.

The Kenya study area has been a Health and Demographic Surveillance System since 2001, covering approximately 227,000 people and 70,000 households. Households are visited three times per year, and a network of "village reporters" provides information on deaths as they occur in the community. Verbal autopsies (using INTER-VA).The study area is served by over 30 health facilities, primarily government and faith-based health centres and dispensaries but also including Siaya District Hospital. The nearest facility capable of conducting autopsies is the regional referral hospital located in Kisumu town, thus the procedure is exceedingly rare and is unfamiliar to most residents.

Mali: Mali is a land-locked country in sub-Saharan Africa ranked with the second highest under-5 mortality rate in the world (178 per 1,000 live births). In 2001, the Centre pour les Vaccins en Développement (CVD-Mali) was established by a formal agreement between the University of Maryland - School of Medicine and the Malian Ministry of Health. Our study will be done in metropolitan Bamako, capital of Mali in the South with ~2 million inhabitants. There about 10 main ethnic groups in Bamako and official language is French and the national language is Bambara. The literacy rate in Bamako is ~ 35%. The District of Bamako has 6 communes and one main paediatric hospital where CVD-Mali team based 24 hours a day and 7 days a week. CVD-Mali has a demographic surveillance going on since 2005 in 2 large quartiers in Bamako.

Mozambique: In Mozambique, the major component of the study will take place in the rural district of Manhiça, Southern part of the country. The district covers an area of 2,360 Km2 and is populated by around 160,000 inhabitants. Part of the district's population live in the Manhiça town centre, and semi-rural neighbourhoods, and the remaining population is scattered around rural villages and administrative posts. They are mostly subsistence farmers, employees of the local sugar processing industry, informal traders and migrant workers in South Africa, Swaziland and Maputo City. Illiteracy rate among adults was 78% in 2008, being more prevalent among women. Manhiça represents the Changanas, which constitute the dominant Ethnic group in Southern Mozambique, with very strong patriarchal social structures and cultural aspects that are similar to other ethnic groups within the southern region of Africa. Christianity and local traditional beliefs are the main belief systems in this area. A Demographic Surveillance System (DSS) has been run by CISM since 1996, covering a population of around 90,000 inhabitants, within an area of around 500 km^2.

Another component of the study will involve participants from Maputo, the country's capital city. It is an urban centre with a population of approximately 2,000,000. However, the majority of the population lives in the peri-urban areas. Participants will be recruited from the Central Hospital, a quaternary level referral facility where CDA occur routinely. There is a mix of ethnic, religious and social backgrounds among the population of Maputo.

Pakistan: In Pakistan, the study will be conducted in Karachi for death occurring outside health system and also in two hospitals; the Aga Khan University Hospital (AKUH) and the Jinnah Postgraduate Medical Center (JPMC) for the deaths occurring within the health system.

Karachi is the most populous city of Pakistan and its main seaport, industrial and financial hub as well as the capital of Sindh province. The city has an estimated population of 23.5 million people as of May 2013, and a density of nearly 6000 people/sq.km. Karachi is the 3rd largest city in the world by population within city limits and 11th largest urban agglomeration. It is Pakistan's center of banking, industry, economic activity and trade and is home to Pakistan's largest corporations, including those involved in textiles, shipping, automotive industry, entertainment, the arts, fashion, advertising, publishing, software development and medical research. The city is a hub of higher education in South Asia and the Muslim world. The city is located in the south of the country, along the coastline meeting the Arabian Sea spread over 4,012 km².

6.2 Target Population

The target population for this study will comprise people or entities that can best describe the phenomenon under study either because they are part of it, affected by it or can affect or influence those who are part of it.

We have divided the target population into 3 groups according to their specific role in the responding to death in the community, as described below.

6.2.1 Key Informants

A key informant has been defined as:

a) Someone who has the privilege to know the community, and/ or can influence the opinion of the community regarding the phenomenon of "death"

- Heads of compounds/ households
- *Family decision makers*
- Local authorities/ Community leaders
- Religious leaders
- Associations/ NGOs / CBOs
- Teachers
- Legal experts
- Community Advisory Boards/ local health committees

b) Someone who knows the rituals and ethnic/ religious norms and requirements for death-related events

- Funeral home personnel
- Religious leaders
- Community elders
- Professional mourners – People who often attend many funerals and are unrelated to the deceased. They help and facilitate the family members to mourn.
- Professionals that manage the corpse: Undertakers, body washers, etc.
- Traditional healers

c) Other entities that should be affected in a second phase study, and not represented in the community of study

- Professionals whose work can be affected by the implementation of MIA (e.g. verbal autopsy interviewers)
- Policy makers
- Governmental authorities

6.2.2 Next of kin of the deceased

The next of kin are defined as the closest possible relative to the deceased, not necessary legally related to the deceased, and with decision making power on family health and death issues, i.e. somebody naturally appointed by the family.

This group of participants will be divided into 2 sub-categories:

a) Those who have suffered a very recent death (within days of the death)

b) Those who have suffered a death between 30-40 days earlier

6.2.3 Health systems professionals

A key health systems professional has been defined as:

a) A health care provider who are regularly in contact with death, specifically at the time of death
   - Family physicians,
   - Nurses
   - Midwives and Traditional Birth Attendants (TBA)
   - Lady Health Visitors/Lady Health Workers/(LHW)/ Community Health Workers (CHW)
   - Traditional healers

b) Professionals in regular contact with death
   - Pathologists
   - Assistants

7. METHODOLOGY

7.1 Recruitment strategy

7.1.1 Rapport building with the community

Before the commencement of the study (and then, throughout the study), the research team will seek permission from the community opinion leaders as well as constituted authorities of the designated study sites (such as the Community Advisory Boards (CABs). Members of the community will be appropriately mobilized and motivated to participate in the study through multiple site-specific mechanisms such as information provision at religious, social, and cultural venues and gatherings and health facilities within the communities. A designated Community Liaison Officer will be dedicated to coordinate all community mobilization activities.

7.1.2 Enrolment

The study will follow two enrolment strategies: one based in the community and the other based in the hospital, for the sites where CDA occur.

Being an ethnographic study, the enrolment approach will be mainly by convenience, i.e. whenever the research team finds an opportunity to approach a potential participant with criteria and willing to share his/her experience. Snow-ball sampling will complement the process, through which further participants will be drawn from networks and contacts of initial participants.

In order to make the above recruitment and enrolment strategies more efficient, each study team will have as members 2 to 3 “ethnographers” who will be locally recruited and specifically train to live or spend substantial and reasonable time in the communities where the study will take place and conduct participant observation.

7.2 Sampling

According to the study needs, subjects will be enrolled according to the following inclusion and exclusion criteria:

- Only those who are willing to talk in some detail about their experiences will be enrolled
- In all violent and accidental deaths, all questions related with the cause of death addressed to the next of kind will be excluded

Sample Size: All the numbers shown below have been established as a minimum. The data collection will continue until the saturation point is reached. This point may be reached before or after the suggested sample size is achieved. See table 1.

**Key informants**	**30 participants**
a) Someone who has the privilege to know the community, and/ or can influence the opinion of the community regarding the phenomenon of "death"	15 participants
b) Someone who knows the rituals and ethnic/ religious norms and requirements for death-related events	10 participants
c) Other entities that should be affected in a second phase study, and not represented in the community of study	5 participants
**Next of Kin**	**30 participants**
a) Those who have suffered a death between 30-40 days earlier	20 participants
· Maternal deaths	4 participants
· Stillborn	2 participants
· Children	2 participants
· Adult	6 participants
· Elders	6 participants
b) Those who have suffered a very recent death (within days of the death)	10 participants
· Maternal deaths	2 participants
· Stillborn	1 participant
· Children	1 participant
· Adult	3 participants
· Elders	3 participants
**Health Systems professionals**	**30 participants**
c) A health care provider who are regularly in contact with death, specifically at the time of death	25 participants
b) Professionals in regular contact with death	5 participants

Table 1. Sample size by Target population groups

7.3 Data collection approaches

7.3.1 Community-Based Data Collection

In-depth Interviews with key informants in order to elucidate cultural, social and religious norms and conduct around community deaths, and to explore informants roles in the local

processes surrounding death and their opinions about performing MIA to deceased individuals of different ages and the best way to proceed if MIA were to be offered.

Focus group discussions with key informants. When required and/or appropriate, the above information will be collected through focus group discussion, because there may be instances when participants might find more comfortable discussing the topic in a group (ex: natural groups such as CABs and community-based associations)

Participant observation of death rituals: Designated members of the social sciences local team will contact and ask permission to accompany the procedures, rituals, customs and traditions around death at the community (health centres, funeral homes, religion services, funerals, etc.) in order to explore attitudes, behaviours and relationships in this context and to understand the local norms and conducts around death. This approach will help to elucidate appropriate ways of enrolling and involving potential participants.

Informal conversations with next-of-kin: Next-of kin of people who have just died (in the last few days or week) will be approached respectfully for an informal and short conversation about the ongoing process of vigil, mourning, their willingness to know the cause of death and the hypothetical acceptability or refusal of the MIA procedure in order to know the attitudes and perceptions immediately after a death.

In-depth interviews with next-of-kin: Family members of recently deceased people (individuals who have suffered a death within the family in the last 30-40 days) will be invited to take part in an interview. Ideally, interviews should be individual but group interviews will also be allowed. Interviewees will be encouraged to give details about the process of vigil, mourning and burial and their opinion on the practice of MIAS. Factors influencing their decision to accept or not to accept the procedure will be identified. Perceived advantages, disadvantages, and consequences of the procedures from the social, cultural, practical, and other perspectives will be discussed. The willingness to know the cause of death will be further explored.

Semi-structured interviews with health professionals: Health workers, especially those who have contact and work based in the communities where autopsies are not regularly performed (e.g. family physicians, investigators, traditional healers, community health workers) will be invited for a semi-structured interview, to understand their own perception of a future intervention requiring the performance of MIAs, their views on the procedures and implications, especially taking into account their own experience in dealing with the family members of the deceased.

7.3.2 Hospital-Based Data Collection:

This aspect will be explored only in Maputo (Mozambique) and those sites that will have access to facilities where autopsies are performed and want to/can include it in the study.

Direct observation

a) ***Observation of the informed consent process:*** In order to gain direct insights of interactions between health workers and family members of the deceased as well as their attitudes and coping strategies when facing the task of asking/ giving consent to perform autopsies (and MIA), a skilled social scientist will be present to observe the whole informed consent process. There will be no interaction between the social scientist and the health worker or with the family members in order to minimize interferences with the decision-making process.

b) ***Observation of the routine study procedures:*** Hospital health workers involved in the MIA study will be under observation while performing their routine activities (including autopsies) in order to determine which procedures are acceptable by them and which strategies and approaches are the most appropriate for a future implementation of MIAs technique.

In depth Interviews from family members of the deceased: 30-40 days after invitation to the main study, family members of the deceased that undergone autopsies and MIAs will be invited to take part in the qualitative study. Interviewees will be encouraged to give details about their experience in taking part in the study and factors influencing their decision to accept or not to accept the procedures will be identified. To those who will have accepted the autopsies (and MIAs), perceived advantages, disadvantages, and consequences of the procedures from the social, cultural, practical, and other perspectives will be discussed.

Semi-structured interviews with hospital health workers: All health workers (nurses, lab technicians, pathologists, investigators) that will subsequently be involved in the main study will be invited for a semi-structured interview once the study is finished, to understand their own perception of the study, the procedures and their experience with dealing with the family members of the deceased.

8. Analysis

Data management and analysis will be conducted using NVivo version 10, software that facilitates the management and coding of large sets of qualitative data.

Audio contents of in-depth interviews, semi-structured interviews and focus group discussions will be digitally recorded, and later on transcribed.

Transcripts, observation reports and field notes will be coded locally by the research team, which will work collaboratively across the sites to develop the coding frame. A generic outline of nodes and codes will be developed (coding tree) which will have the flexibility of including emerging themes from specific sites. As the emerging themes are incorporated, they will be shared with the investigators of the 4 sites and in that way the coding tree will be continuously updated. Coded text will be translated and shared with the other sites for multisite analysis.

A descriptive analysis will be performed for all quantitative indicators (ex: quantifiable variables from the semi-structured interviews) by frequency distribution.

9. Ethical Considerations

Informed consent will be read out and a copy of the study information sheet will be handed to each participant. Participation in the feasibility study will be voluntary, and confidentiality will be preserved in accordance with the national legislation regarding data protection, or in the absence of this, in accordance with the GCP ICH norms.

Notes and documents containing identification data will be only accessible to the staff members that deal with the participants. The remaining members of the team (data analysts and other personnel authorised by the PI) will only have access to the contents of the interviews and the participants ID numbers.

10. References

[i] Fligner CL, Murray J, Roberts DJ. Synergism of verbal autopsy and diagnostic pathology autopsy for improved accuracy of mortality data. Popul Health Metr 2011;9:25.
[ii] Tsitsikas DA, Brothwell M, Aleong JAC, et al. The attitudes of relatives to autopsy: a misconception. J Clin Pathol 2011; 64:412-4
[iii] Oluwasola OA, Fawole OI, Otegbayo AJ, et al. The Autopsy. Knowledge, Attitude and Perceptions of Doctors and Relatives of the Deceassed. Arch Pathol Lab med 2009; 133:78-82
[iv] El-Reshaid W, El-Reshaid K, Madda J. Postmortem Biopsies: The experience in Kuwait. Med Princ Pract 2005; 14:173-6
[v] Waldron G, Morgan R, Raffles A, Keen CE, Meadow R, Rahman HA. Perinatal and infant post-mortem examination [editorial]. BMJ 1995; 310:870-1
[vi] Ordi J, Ismail MR, Carrilho C, et al. Clinico-pathological discrepancies in the diagnosis of causes of maternal death in sub-Saharan Africa: retrospective analysis. PLoS Med 2009;6:e1000036.
[vii] Taylor TE, Fu WJ, Carr RA, et al. Differentiating the pathologies of cerebral malaria by postmortem parasite counts. Nat Med 2004;10:143-5
[viii] Ugiagbe EE, Osifo OD. Postmortem Examinations on Deceased Neonates: A Rarely Utilized Procedure in an African Referral Center. Pediatr Dev Pathol 2011
[ix] Chintu C, Mudenda V, Lucas S, et al. Lung diseases at necropsy in African children dying from respiratory illnesses: a descriptive necropsy study. Lancet 2002;360:985-90
[x] Breeze AC, Jessop FA, Set PA, et al. Minimally-invasive fetal autopsy using magnetic resonance imaging and percutaneous organ biopsies: clinical value and comparison to conventional autopsy. Ultrasound Obstet Gynecol 2010;37:317-23.
[xi] Breeze AC, Jessop FA, Whitehead AL, et al. Feasibility of percutaneous organ biopsy as part of a minimally invasive perinatal autopsy. Virchows Arch 2008;452:201-7.
[xii] Sebire NJ. Towards the minimally invasive autopsy? Ultrasound Obstet Gynecol 2006;28:865-7.
[xiii] Sebire NJ, Weber MA, Thayyil S, Mushtaq I, Taylor A, Chitty LS. Minimally invasive perinatal autopsies using magnetic resonance imaging and endoscopic postmortem examination ("keyhole autopsy"): feasibility and initial experience. J Matern Fetal Neonatal Med 2012;25:513-8.
[xiv] Thayyil S, Sebire NJ, Chitty LS, et al. Post mortem magnetic resonance imaging in the fetus, infant and child: A comparative study with conventional autopsy (MaRIAS Protocol). BMC Pediatr 2011;11:120.
[xv] McBeth PB, Crawford I, Blaivas M, et al. Simple, almost anywhere, with almost anyone: remote low-cost telementored resuscitative lung ultrasound. The Journal of trauma 2011;71:1528-35

Annex 1: Timeline:

ACTIVITIES	2013										2014												2015											
	MAR	APR	MAY	JUN	JUL	AUG	SEP	OCT	NOV	DEC	JAN	FEB	MAR	APR	MAY	JUN	JUL	AUG	SEP	OCT	NOV	DEC	JAN	FEB	MAR	APR	MAY	JUN	JUL	AUG	SEP	OCT	NOV	DEC
Investigators Meeting	■																								■									
Preparation: Protocols; tool development; ethics clearance		■	■	■	■	■	■																											
Team members: Recruitment; training					■	■	■																											
Data collection							■	■	■	■	■	■	■	■	■	■	■	■	■															
Coding								■	■	■	■	■	■	■	■	■	■	■	■	■	■	■												
Final analysis output																						■	■	■	■	■	■							
Final report																											■	■	■	■				

Annex 2: Budget

	Budget 2013	Budget 2014	Budget 2015	Total Budget
**Project management - Personnel**	**$10.000**	**$10.000**	**$5.000**	**$25.000**
Name 1				
Name 2				
…				
**Objective 2 - Social feasibility**	**$15.000**	**$25.000**	**$0**	**$40.000**
Transcription kits (2 u.)	$500	$0	$0	$500
Digital recorders (2 u.)	$500	$0	$0	$500
Office supplies + fieldw ork material	$1.200	$2.000	$0	$3.200
Nvivo softw are license	$1.300	$1.300	$0	$2.600
Transcriptional services	$10.000	$20.000	$0	$30.000
Official procedures	$500	$0	$0	$500
Field transport & shipment	$1.000	$1.700	$0	$2.700
**TOTAL DIRECT COSTS**	**$25.000**	**$35.000**	**$5.000**	**$65.000**
**TOTAL INDIRECT COSTS**	**$3.750**	**$5.250**	**$750**	**$9.750**
**GRAND TOTAL COSTS**	**$28.750**	**$40.250**	**$5.750**	**$74.750**

Annex 3: Informed Consent

INFORMED CONSENT
CaDMIA Feasibility and Acceptability Study

Validation of a sample collection tool to investigate the cause of death in developing countries

INVESTIGATORS: Dr Quique Bassat, Overall Principal Investigator
Dr Clara Menéndez, Overall Project Co-Principal Investigator

Barcelona Center for International Health Research (CRESIB), Spain

Dr Khátia Munguambe, Feasibility and Acceptability Study Principal Investigator

Centro de Investigação em Saúde da Manhiça (CISM), Mozambique

Site Institution: [Each site to complete]

Local Principal Investigator: [Each site to complete]

Local Social Sciences Investigators: [Each site to complete]

Sponsor: Barcelona Center for International Health Research - CRESIB

Purpose of the Study
We (Each site Institution's name) are conducting the CaDMIA Feasibility and Acceptability study. This study is about a new sample collection tool to investigate the cause of death called MIA. MIA is a tool that uses a needle puncture to extract a small sample of an organ's tissue. This small sample is examined in a laboratory to see if it can help us to learn the cause of death of the person. No large cuts are needed and no organs are removed.

This study is being conducted in Mozambique, Kenya, Gabon, Mali and Pakistan.

We are asking you to participate in the interview / group discussion so we can learn about what happens at the community and within the family when somebody dies and all the practices and rituals around death. We are also interested about the interaction within the health system (hospital, clinic, etc.) when a death happens. We want to explore if people are willing to know the cause of death of a loved one and we are interested to hear your thoughts about the benefits or any concerns you may have about the possible future use of the MIA tool in your community.

Type of Study

This study will involve your participation in an interview/discussion. It will take about 1-2 hours. We are asking you to participate in the interview in order to provide information and share your knowledge about practices, norms and experiences around death and about the benefits and concerns of the MIA tool.

Voluntary Participation

Your participation in this interview is entirely voluntary. It is your choice whether to participate or not. There will be no penalty or loss of benefits to which you are otherwise entitled and your

choice will not affect your position in anyway. You may change your mind later and stop participating at any time even if you agreed earlier or during the course of questioning. You can withdraw from the study at any time.

Potential Risks of Discomfort

There are no possible risks involved in the study. You do not have to answer any question or take part in the discussion if you feel the question(s) are too personal or if talking about them makes you uncomfortable. Photographs may be taken during this discussion or interview; these will be used for scientific purposes only and will not contain any other personal information. You can decline to have photographs taken if you wish.

Benefits

The MIA tool could improve knowledge about cause of death in this community. This could help improve health programs and use of health resources in your community. Your participation will help us to learn more about how to implement the MIA tool, if possible, and the better way to do it.

Reimbursements

You will not receive any monetary reimbursements for participating in the study; your participation will be voluntary.

Confidentiality

Your confidentiality will be respected. No information that discloses your identity will be released. All the information taken through interviews with you will be kept safely and no person other than authorized local key investigators will be able to trace the information to your name or your address. The data with all identification removed will be shared with other scientists working on this project; this means that the words will be shared but not who said them. Any names you mention will be removed.

Whom to contact with questions about the study

We have given you information about the study called "The CaDMIA feasibility and acceptability study." We have discussed the risks and benefits of the study and you understand that you do not have to agree to be in the study or may decide later not to be part of the study.

If you have any questions, please call:
Dr. [Name] at [Telephone number], Mobile No: [Mobile number]
Dr. [Name] at [Telephone number], Mobile No: [Mobile number]
Dr. [Name] at [Telephone number], Mobile No: [Mobile number]

If you have questions about your rights as a study participant, please contact Dr [Name], Chairman, [Name of each Country Ethics Committee] at [Telephone number]

I have read this consent form or it has been read to me in presence of a witness in my vernacular. I was given opportunity to ask questions and they were answered to my satisfaction. By signing this document I declare that I have consented to participate in this study.

____

Signature or Thumbprint of Person Providing Consent Date

____

Signature or Thumbprint of Witness Date

____

Signature of Person Obtaining Consent Date

Annex 4: Guides

1. Key Informants Interview Guide

Introduction

I am ___________________ from [Name of each Site]. I welcome you in this interview. I will be the moderator for this interview and I will be taking notes and recording audio during this discussion.

Purpose

This interview aims to explore your knowledge, beliefs and attitudes about deaths occurring at the community, the significance to know the cause of death and the possibility of performing MIAs.

You have been invited for this interview because your input is essential in understanding the phenomenon of death in your community. We will initiate our discussion with general questions about your knowledge of what happens when death occur, we will then talk about issues related to the cause of death and the possibility of the performance of MIAs in your community.

This interview is expected to take 1 – 1.5 hours.

Ground Rules

We will be recording this interview to ensure we do not miss any of the responses or discussion. I will also be taking notes throughout the discussion for backup. All information recorded will be kept confidential and you will not be identified by name. You may chose not to respond at any time.

Themes:

ICE BREAKER: What is the respondent role in the process (around death)?

THEME I: What happens when somebody dies

- Chronology of events
- People interacting with the body
- Burial process
- Mourning “process”
- Cultural and religious beliefs around death
- Cost of death
- Which is the person to approach within the family?

THEME II: Cause of Death

- Would the community see any benefit/ value in knowing the cause of death?

THEME III: MIA

- Would it be acceptable to perform any procedure to the deceased body? Will make a difference if the procedures are addressed to elders /adults /pregnant women/ children/ stillborn deceased body?
- Where MIA would be ideally performed?
- What kind of incentive/compensation (if any) would be required?
- How would MIA be named in your community?

END OF THE INTERVIEW

CLOSING COMMENTS:

I am very thankful for agreeing to participate in this important discussion and valuable time that you spared. Your comments are very important to guide us in future interventions to better understand the causes of death in our country. Please feel free to ask me any questions you may have.

2. Key Informants Focus Group Discussions Guide

Introduction

I am ___________________ from [Name of each Site]. I welcome you in this focus group discussion. I will be the moderator for this focus group and with me Mr/Ms. _______________ from [Name of each Site] will be taking notes and recording audio during this discussion.

Purpose

This focus group aims to explore your knowledge, beliefs and attitudes about deaths occurring at the community, the significance to know the cause of death and the possibility of performing MIAs.

You have been invited for this group discussion because your input is essential in understanding the phenomenon of death in your community. We will initiate our discussion with general questions about your knowledge of what happens when death occur, we will then talk about issues related to the cause of death and the possibility of the performance of MIAs in your community.

This focus group is expected to take 1 – 1.5 hours.

Ground Rules

We will be recording this focus group to ensure we do not miss any of the responses or discussion. The note taker will also be taking notes throughout the discussion for backup. All information recorded will be kept confidential and you will not be identified by name. You may chose not to respond at any time.

In order to promote group cohesion and giving everybody equal chance to speak, we will follow group rules:

- All participants will have the chance to respond if desired
- All participants will wait for their turn to speak
- All participants will respect each other's' point of view

Themes

ICE BREAKER: What is the respondents' role in the process (around death)?

THEME I: What happens when somebody dies

- Chronology of events
- People interacting with the body
- Burial process
- Mourning "process"
- Cultural and religious beliefs around death
- Cost of death
- Which is the person to approach within the family?

THEME II: Cause of Death

- Would the community see any benefit/ value in knowing the cause of death?

THEME III: MIA

- Would it be acceptable to perform any procedure to the deceased body? Will make a difference if the procedures are addressed to elders /adults /pregnant women/ children/ stillborn deceased body?
- Where the MIA would be ideally performed?
- What kind of incentive/compensation (if any) would be required?
- How would MIA be named in your community?

END OF FOCUS GROUP

CLOSING COMMENTS:

I am very thankful for agreeing to participate in this important discussion and valuable time that you spared. Your comments are very important to guide us in future interventions to better understand the causes of death in our country. Please feel free to ask me any questions you may have.

3. Next of Kin Interview Guide

Introduction

I am ____________________ from [Name of each Site]. I welcome you in this interview. I will be the moderator for this interview and I will be taking notes and recording audio during this discussion.

Purpose

This interview aims to explore your experience during your recent lost. You have been invited for this interview because your input is essential in understanding how people manage when a death occurs within the family. We will initiate our discussion with general questions about your recent experience with death. We will then talk about the significance to know the cause of death and your opinion about the possibility of performing of MIAs.

This interview is expected to take 1 – 1.5 hours.

Ground Rules

We will be recording this interview to ensure we do not miss any of the responses or discussion. I will also be taking notes throughout the discussion for backup. All information recorded will be kept confidential and you will not be identified by name. You may chose not to respond at any time.

Themes

THEME I: Death

- Decision making process
- Experiences and problems during the process
- Trust on and interaction with health professionals and funeral homes

THEME II: Cause of death

- Cause of death relevance

THEME III: MIA

- Benefits and concerns of MIA
  - Fears about managing the corpse and sampling

- Particular concerns for each group
  - Elders
  - Adults
  - Maternal deaths (if possible)
  - Children
  - Stillborns (if possible)

END OF THE INTERVIEW

CLOSING COMMENTS

I am very thankful for agreeing to participate in this important discussion and valuable time that you spared. Your comments are very important to guide us in future interventions to better understand the causes of death in our country. Please feel free to ask me any questions you may have.

4. Health systems professionals Interview Guide

Introduction

I am ___________________ from [Name of each Site]. I welcome you in this interview. I will be the moderator for this interview and I will be taking notes and recording audio during this discussion.

Purpose

This interview aims to explore your knowledge, beliefs and attitudes about deaths occurring at your place of work, the significance to know the cause of death and the possibility of performing MIAs.

You have been invited for this interview because your input is essential in understanding the processes around death. We will initiate our discussion with general questions about the procedure of death; we will then talk about the experience of dealing with the family members of the deceased, the significance to know the cause of death and the and the possibility of performing MIAs.

This interview is expected to take 1 – 1.5 hours.

Ground Rules

We will be recording this interview to ensure we do not miss any of the responses or discussion. I will also be taking notes throughout the discussion for backup. All information recorded will be kept confidential and you will not be identified by name. You may chose not to respond at any time.

Themes

ICE BREAKER: What is the respondent role in the process (around death)?

THEME I: Procedure for death

- Informing (official and family)
- Handling and care of the body
- Legal aspects

THEME II: Experiences dealing with death and communicating death to family members

THEME II: Cause of death and MIA

- Professional concerns about knowing the cause of death and about autopsy / MIA
  - Value & Benefits
  - Determination of CoD process

END OF THE INTERVIEW

CLOSING COMMENTS:

I am very thankful for agreeing to participate in this important discussion and valuable time that you spared. Your comments are very important to guide us in future interventions to better understand the causes of death in our country. Please feel free to ask me any questions you may have.
